# *Tbx1*, a gene encoded in 22q11.2 copy number variant, is a link between alterations in fimbria myelination and cognitive speed in mice

**DOI:** 10.1038/s41380-021-01318-4

**Published:** 2021-11-05

**Authors:** Takeshi Hiramoto, Akira Sumiyoshi, Takahira Yamauchi, Kenji Tanigaki, Qian Shi, Gina Kang, Rie Ryoke, Hiroi Nonaka, Shingo Enomoto, Takeshi Izumi, Manzoor A. Bhat, Ryuta Kawashima, Noboru Hiroi

**Affiliations:** 1 Department of Pharmacology, University of Texas Health Science Center at San Antonio, San Antonio, TX 78229 USA; 2 Institute of Development, Aging, and Cancer, Tohoku University, 4-1, Seiryo-cho, Aoba-ku, Sendai, 980-8575 Japan; 3 National Institutes for Quantum and Radiological Science and Technology, 4-9-1, Anagawa, Inage-ku, Chiba 263-8555 Japan; 4 Research Institute, Shiga Medical Center, 5-4-30 Moriyama, Moriyama-shi, Shiga Japan; 5 Department of Cellular and Integrative Physiology, University of Texas Health Science Center at San Antonio, San Antonio, TX 78229 USA; 6 Department of Psychiatry and Behavioral Sciences, Albert Einstein College of Medicine, 1300 Morris Park Avenue, Bronx, NY 10461 USA; 7 Department of Pharmacology, Health Sciences University of Hokkaido, 1757 Kanazawa, Tobetsu, Ishikari, Hokkaido 061-0293 Japan; 8 Advanced Research Promotion Center, Health Sciences University of Hokkaido, 1757 Kanazawa, Tobetsu, Ishikari, Hokkaido 061-0293 Japan; 9 Department of Cell Systems and Anatomy, University of Texas Health Science Center at San Antonio, San Antonio, TX 78229 USA

**Keywords:** Autism spectrum disorders, Neuroscience

## Abstract

Copy number variants (CNVs) have provided a reliable entry point to identify the structural correlates of atypical cognitive development. Hemizygous deletion of human chromosome 22q11.2 is associated with impaired cognitive function; however, the mechanisms by which the CNVs contribute to cognitive deficits via diverse structural alterations in the brain remain unclear. This study aimed to determine the cellular basis of the link between alterations in brain structure and cognitive functions in mice with a heterozygous deletion of *Tbx1*, one of the 22q11.2-encoded genes. Ex vivo whole-brain diffusion-tensor imaging (DTI)–magnetic resonance imaging (MRI) in *Tbx1* heterozygous mice indicated that the fimbria was the only region with significant myelin alteration. Electron microscopic and histological analyses showed that *Tbx1* heterozygous mice exhibited an apparent absence of large myelinated axons and thicker myelin in medium axons in the fimbria, resulting in an overall decrease in myelin. The fimbria of *Tbx1* heterozygous mice showed reduced mRNA levels of *Ng2*, a gene required to produce oligodendrocyte precursor cells. Moreover, postnatal progenitor cells derived from the subventricular zone, a source of oligodendrocytes in the fimbria, produced fewer oligodendrocytes in vitro. Behavioral analyses of these mice showed selectively slower acquisition of spatial memory and cognitive flexibility with no effects on their accuracy or sensory or motor capacities. Our findings provide a genetic and cellular basis for the compromised cognitive speed in patients with 22q11.2 hemizygous deletion.

## Introduction

Although copy number variants (CNVs) are rare and occur in <1% of patients with any psychiatric sample population, they are robustly and consistently associated with psychiatric disorders [1, 2]. Moreover, CNVs affect specific cognitive functions independent of clinically defined mental illness [3, 4] and cognitive impairments are more severe among CNV carriers with psychiatric diagnosis [5]. The currently available pharmaceutical medications do not significantly improve cognitive deficits associated with many mental disorders due to our lack of understanding of the mechanisms underlying these deficits.

Despite their robust association with cognitive impairments and psychiatric disorders, CNVs pose a challenge when attempting to understand the composition of contributory genes, as accurate identification of CNV-encoded genes contributing to human phenotypes remains difficult. Recent large-scale genome-wide exome screening studies have identified ultra rare protein-truncating variants of genes encoded in several large-sized CNVs linked with autism-spectrum disorder (ASD) [6] and schizophrenia [7]. However, failure to detect similar variants of other CNV-encoded genes may be attributable to their rarity, as larger sample sizes identify more gene variants than smaller-scale analyses [6]. Variants in promoters and enhancers may contribute to phenotypes [8]. Moreover, variants of CNV-encoded single genes may simply not exist, and single-gene hemizygosity or duplication, as part of a CNV, may play the role of a driver gene. Thus, there is a need to utilize complementary approaches to identify driver genes encoded by large CNVs.

There are more human and mouse studies of human chromosome 22q11.2 deletion than other CNVs, given that it was found to be associated with mental illness much earlier than other CNVs [9]. Hemizygous deletion of 22q11.2 is robustly associated with diverse neurodevelopmental disorders, including ASD, attention-deficit/hyperactivity disorder, schizophrenia, and intellectual disability (ID) [10]. Moreover, individuals with 22q11.2 hemizygosity exhibit deterioration in specific cognitive functions, including the accuracy and speed of memory acquisition, executive functions, and social cognition [11–13]. Cognitive impairment may be an integral component of late-onset neurodevelopmental disorders, as it precedes and predicts the onset of schizophrenia among 22q11.2 hemizygosity carriers [14, 15].

Recent large-scale imaging studies have demonstrated altered white matter integrity in the brains of 22q11.2 hemizygous deletion carriers [16–18]. However, since many regions show altered white matter integrity and this CNV contains a minimum of 30 protein-coding genes, the exact causative associations among encoded genes, structural alterations, and atypical cognitive development remain unclear.

Rare loss-of-function variants (e.g., frameshift deletion) of *TBX1*, a gene encoded by a 22q11.2 CNV, have been associated with ASD, ID, and seizures [19–22]. However, these *TBX1* variant carriers also exhibit single-nucleotide variants (SNVs) in other genes [21], and only a few cases/families with those variants have been identified. A recent large-scale exome sequencing identified an ultra rare protein-truncating variant of *TBX1* among individuals with schizophrenia [7], but its statistical significance is unclear due to power limitation. The causative structural substrates in the brain mediating the impacts of deficiency of this 22q11.2-encoded gene on cognitive impairment remain unknown.

Mouse studies have provided a complementary means to address limitations of these human studies by systematically examining the roles of small chromosomal segments and individual genes in behaviors against a homogeneous genetic background [10, 22–32]. These studies have demonstrated that some, but not all, 22q11.2-encoded single genes contribute to select behavioral targets [10, 28, 31]. Our results have revealed that *Tbx1* heterozygosity impairs social interaction and communication [26, 29, 30, 32]. No DTI–MRI analysis of mouse models of 22q11.2 hemizygosity or *Tbx1* heterozygosity has been reported. The present study aimed to determine the structural and cellular basis underlying the effects of *Tbx1* heterozygosity on specific cognitive functions in a congenic mouse model.

## Methods and materials

Animal handling and use followed the protocols that were approved by the Animal Care and Use Committees of Albert Einstein College of Medicine and the University of Texas Health Science Center at San Antonio, in accordance with NIH guidelines.

All experimental details are provided in Supplementary Information (SI). Briefly, congenic *Tbx1* heterozygous mice and their wild-type littermates were used. We characterized the myelin in the brains, using ex vivo DTI–MRI, Black Gold II staining, and electron microscopy (EM) analyses. Genes involved in oligodendrocytes and their precursor cells were evaluated by qRT-PCR. The production capacity of oligodendrocyte precursor cells was evaluated in vitro using precursor cells derived from the subventricular zone of postnatal (P21) mice. The accuracy and speed of spatial memory and of cognitive flexibility were evaluated in the Morris water maze and attentional set shifting, respectively. Moreover, nonspecific deficits of visual and olfactory senses and general motivation to approach an object were examined using olfactory responses to nonsocial and social cues. The minimal sample size was determined by power analyses based on our previous study [26].

We compared group means using analysis of variance (ANOVA), followed by Newman–Keuls post hoc tests. Two-sided *t*-tests were used when there were only two groups. A probability of ≤0.05 was considered significant. When either homogeneity of variance or normality was violated, data were analyzed by a generalized linear mixed model; for comparisons of a pair of data, nonparametric tests were used. When multiple tests were applied to a dataset, the significance level was adjusted using Benjamini–Hochberg’s correction.

## Results

There are alterations in white matter microstructures in many brain regions of 22q11.2 hemizygosity carriers [16–18]. However, little is known regarding the exact nature of altered white matter microstructures and driver genes that affect both the white matter and cognitive functions in humans and mice.

### Analysis of white matter structures

#### *Tbx1* deficiency decreases fractional anisotropy (FA) signals in the fimbria

**Fig. 1:**
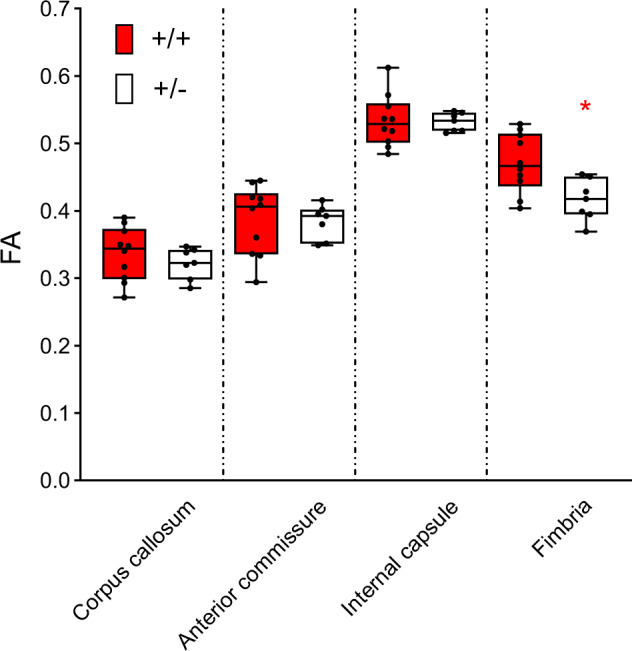
Box-and-whisker plots of fractional anisotropy (FA) values of the four regions with FA >0.3. Analysis using a generalized linear mixed model revealed a region-dependent differential effect of genotype on FA values (Genotype × Region, F(3,45) = 7.337, *P* < 0.001). Mann–Whitney U-tests revealed a significant between-genotype difference in the fimbria only (*, U = 11, *p* = 0.0185). +/+, *n* = 10; +/−, *n* = 7.

*Tbx1* +/− mice and their +/+ littermates underwent ex vivo DTI–MRI. We analyzed 19 brain regions (Fig. S1), as defined by the standard regional classification of the mouse brain [33]. The FA value is the most histologically validated DTI–MRI metric [34]. Since FA signals of <0.3 are not reliably correlated with the degree of myelination [35] and cannot be accurately aligned across individual animals [36], we selected regions with FA values ≥0.3. The corpus callosum, anterior commissure, internal capsule, and fimbria met this criterion (Fig. S2a). The fimbria was the only region exhibiting a significant change: FA values were lower in +/− mice than in +/+ mice (Fig. 1). Consistently, the fimbria exhibited the largest effect size for genotype-dependent differences in FA values (Fig. S2b). There were no significant differences in axial diffusivity (AD) (Fig. S3a, b), radial diffusivity (RD) (Fig. S4a, b), or mean diffusivity (MD) (Fig. S5a, b) values between +/+ and +/− mice.

#### *Tbx1* deficiency reduces myelination in the fimbria

DTI–MRI analysis of the mouse brain has limited spatial resolution, as well as technical and interpretative limitations [34, 37]. Our resolution (150 μm isotropic voxel) may not have been sufficient for detecting subtle alterations. The structural classifications of the mouse brain by Ma et al. [33] include the fimbria, fornix, stria terminalis, and hippocampal commissure in the “fimbria”. To circumvent these limitations and histologically validate the DTI–MRI findings, we used the non-hydroscopic gold-phosphate complex Black-Gold II [38]. This method provides more consistent staining than hydroscopic gold chloride staining and higher contrast and resolution than lipid-soluble dyes (e.g., Luxol Fast Blue). Black-Gold II also directly stains myelin, unlike markers of myelin components (e.g., myelin basic protein [MBP]), which may not perfectly correlate with the degree of myelination. We examined regions with the largest and second-largest effect sizes among FA values >0.3 (see Fig. S2b): the fimbria and corpus callosum (Fig. S6). The intensity of gold staining was lower in the anterior fimbria of +/− mice than in that of +/+ mice (Fig. S7a). There was no statistically detectable between-genotype difference in the posterior fimbria or anterior/posterior isthmus of the corpus callosum (Fig. S7b,c,d).

#### *Tbx1* deficiency reduces large myelinated axons in the fimbria

**Fig. 2:**
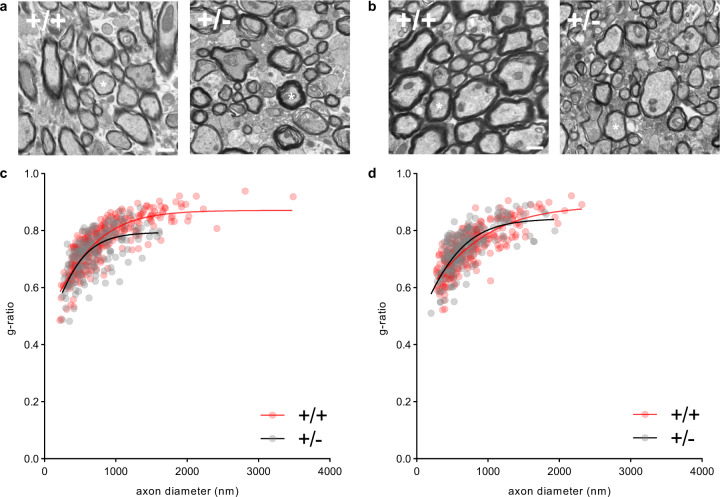
EM analysis of myelinated axons. EM images of myelin in the fimbria (**a**) and corpus callosum (**b**) are shown. We analyzed 300 and 200 axons in the fimbria of both hemispheres in three +/+ and two +/− mice, respectively. We analyzed 260 and 200 axons in the corpus callosum of both hemispheres in three +/+ and two +/− mice, respectively. Ten images were obtained from the fimbria or corpus callosum of each mouse, except for one +/+ mouse that had six available images of the corpus callosum. Ten randomly chosen myelinated axons were analyzed from each image. Scale bar = 800 nm. G ratios increased as a logarithmic function of axon diameter in the fimbria (**c**, +/+, *R* = 0.845, *p* < 0.001; +/−, *R* = 0.686, *p* < 0.001) and corpus callosum (**d**, +/+, *R* = 0.804, *p* < 0.001; +/−, *R* = 0.749, *p* < 0.001). The degree of these increases differed in the fimbria (Fisher’s *z* = 4.3319, *p* < 0.0001), but not in the corpus callosum (Fisher’s *z* = 1.4695, *p* = 0.1417), between +/− and +/+ mice (see Fig. S8 for detailed analyses).

We used electron microscopy (EM) to characterize the myelination of axons in the fimbria and corpus callosum at the ultrastructural level. Myelination appeared thicker and thinner in the fimbria and corpus callosum, respectively, of +/− mice than in that of +/+ mice (Fig. 2a, b). We compared g-ratios (i.e., the ratio of axon diameter to the axon + myelin diameter) to quantitatively evaluate relative myelin thickness (Fig. 2c, d). The g-ratios of +/+ mice plateaued slightly above 0.8, which is an expected value for the optimal efficiency of axon myelination in the central nervous system (CNS) [39]. The g-ratios of +/− mice were smaller (i.e., relatively thicker myelin sheath) between 700-nm and 1600-nm-diameter axons in the fimbria (Fig. 2c; Fig. S8a). The overall g-ratios did not differ in the corpus callosum between +/+ and +/− mice (Fig. 2d), but +/− mice had relatively thinner myelin sheath between 1000-nm and 1300-nm-diameter axons in this region (Fig. S8b). Volume is a limiting factor in the CNS: The myelination efficiency steeply decreases when myelin thickness deviates from the optimal g-ratios (~0.8) [39], regardless of whether it is hyper- or hypomyelination. Therefore, this gene deficiency results in a functionally suboptimal population of axons in the fimbria.

A further in-depth analysis revealed that there were proportionally fewer myelinated axons >1600 nm in diameter in the fimbria of +/− mice than of +/+ mice (Fig. S9a; Table S1) and that there were no detectable myelinated axons >1700 nm in diameter in the fimbria of +/− mice (Fig. S9a**;** Table S1). In the corpus callosum, there were no statistically significant alterations in relative proportion of axons in the corpus callosum of +/− mice (Fig. S9b; Table S1).

#### *Tbx1* heterozygosity impacts a molecule critical for early oligodendrogenesis

**Fig. 3:**
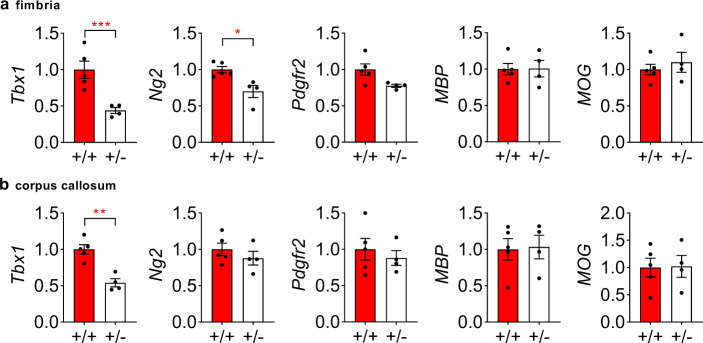
Effect of *Tbx1* heterozygosity on myelin-related genes. Relative mRNA expression levels (mean ± standard error of the mean [SEM]) for *Tbx1*, *Ng2*, *Pdgfr2*, myelin basic protein (MBP), and myelin oligodendrocyte glycoprotein (MOG) in the fimbria (**a**) and corpus callosum (**b**) of *Tbx1*+/+ (*n* = 5) and +/− (*n* = 4) mice are shown. *Tbx1* mRNA levels were lower in the fimbria (**a**, *t*(7) = 4.081, *p* = 0.0047, ***) and corpus callosum (**b**, *t*(7) = 5.221, *p* = 0.0012, **) of +/− mice than in those of +/+ mice. In the fimbria, levels of *Ng2* (**a**, *t*(7) = 3.394, *p* = 0.0115, *) were lower in +/− mice than in +/+ mice. These significant differences survived Benjamini–Hochberg’s correction at the false-discovery rate (FDR) of 5%. There were no other significant differences in the fimbria or corpus callosum (**a,b**, *p* > 0.05).

Oligodendrocytes and their precursor cells are present locally in the fimbria and, to a lesser extent, in the corpus callosum. The molecular steps through which *Tbx1* impacts oligodendrogenesis and myelination remain unknown. Given that *Tbx1* mRNA is reduced in the fimbria and corpus callosum of *Tbx1* +/− mice compared with +/+ mice (Fig. 3), we examined the impact of a gene-dose reduction of *Tbx1* mRNA on genes functionally critical for each step of oligodendrogenesis and myelination in 2- to 3-month-old *Tbx1* +/− and +/+ littermates, using qRT-PCR. The myelinating process—and expression of genes involved in each step of oligodendrocytes—of the fimbria starts in the second neonatal week and continues its peak levels from postnatal day 24 to 37 in rodents [40, 41].

*Ng2* (*Cspg4*) and *Pdgfr2*, markers of oligodendrocyte precursor cells, are functionally required for the production of oligodendrocyte precursor cells [42, 43]. We found that mRNA levels of *Ng2*, but not of *Pdgrf2*, were selectively lower in the fimbria of +/− mice than in that of +/+ mice (Fig. 3a). There was no detectable difference in *Ng2* or *Pdgfr2* mRNA levels in the corpus callosum between +/+ and +/− mice (Fig. 3b). *MBP* is essential for the maintenance of myelin and is involved in the adhesion and compaction of the cytosolic membrane leaflets that form the structural basis of multilayered myelin [44, 45]. Myelin oligodendrocyte glycoprotein (*MOG*) is a marker of mature oligodendrocytes and myelin, although it is not functionally critical for myelin formation or maintenance [46]. No differences in *MBP* or *MOG* levels were observed in the fimbria or corpus callosum between +/+ and +/− mice (Fig. 3ab).

#### *Tbx1* heterozygosity reduces oligodendrocyte generation

**Fig. 4:**
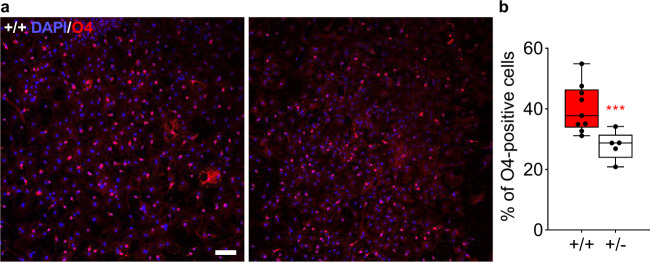
Effect of Tbx1 heterozygosity on oligodendrocytes. Representative images (**a**) and box-and-whisker plots (**b**) of O4-positive (red) oligodendrocytes among all DAPI-positive (blue) cells in culture are shown. Since the assumption of normality was not met (Shapiro–Wilk tests: +/+, W(35) = 0.888, *p* = 0.002; +/−, W(19) = 0.898, *p* = 0.045), we applied a generalized linear mixed model of log-transformed data. Progenitor cells derived from the lateral ventricular walls of P21 *Tbx1* +/− mice produced consistently fewer O4-positive oligodendrocytes than those of +/+ mice across the cultures (Genotype, F(1,11.451) = 12.841. *p* = 0.004, *** Image field, F(3, 33.978) = 0.609, *p* = 0.614; Genotype x Image field, F(3,33.978) = 0.134, *p* = 0.939). Scale bar = 200 μm. +/+, *n* = 9; +/−, *n* = 5.

Another source of oligodendrocytes in the fimbria is the population of adult neural progenitor cells in the subventricular zone [47–50], which is distinct from those generating neurons [51]. Given the enrichment of Tbx1 protein in the adult subventricular zone (SVZ) [26], we aimed to determine whether *Tbx1* heterozygosity affects the cell-autonomous capacity of this oligodendrocyte population. Progenitor cells were taken from the lateral ventricular wall, including the subventricular zone, of 3-week-old *Tbx1* +/− and +/+ littermates and cultured and differentiated into oligodendrocytes in vitro. Progenitor cells derived from the subventricular zone of *Tbx1* +/− mice produced fewer O4-positive immature and mature oligodendrocytes than those derived from +/+ mice (Fig. 4ab). This in vitro assay demonstrated that *Tbx1* heterozygosity reduced oligodendrocyte production from progenitor cells of the SVZ in a cell-autonomous manner.

Although decreased FA values and reduced net myelin signals are suggestive of less myelin, axonal degeneration, reduced axonal density, or changes in axonal organization [52], our EM analyses complemented these assessments of the net signal intensities by demonstrating a loss of large myelinated axons in the fimbria. Our qRT-PCR and in vitro analyses further indicated that *Tbx1* heterozygosity reduced levels of the molecule needed for the generation of local oligodendrocyte precursor cells and the production capacity of oligodendrocytes in the lateral ventricular wall.

### Analysis of cognitive functions

Individuals with 22q11.2 hemizygous deletions exhibit lower scores on measures of attention, executive function, processing speed, visual memory, visuospatial skills, and social cognition [11, 13]. However, the link between structural alterations caused by single 22q11.2 genes and changes in cognitive function remains unknown. In addition, although human studies have reported an association between loss-of-function *TBX1* variants and neurodevelopmental disorders [7, 19–22], their effects on cognitive function remain uncharacterized. Since we observed that *Tbx1* heterozygosity leads to myelin alterations in the fimbria, we examined its effects on cognitive capacities known to rely on the fimbria.

#### *Tbx1* heterozygosity slows the acquisition of spatial reference memory

The spatial reference memory version of the Morris water maze requires an intact fimbria, whereas the visual cued version depends on the dorsal striatum in rodents [53, 54]. Humans with 22q11.2 hemizygosity exhibit impaired spatial processing and memory [11, 55–57]. While our congenic *Tbx1* heterozygous mice are normal in motor capacities [26], the effect of *Tbx1* deficiency on spatial memory has not been examined in mice.

**Fig. 5:**
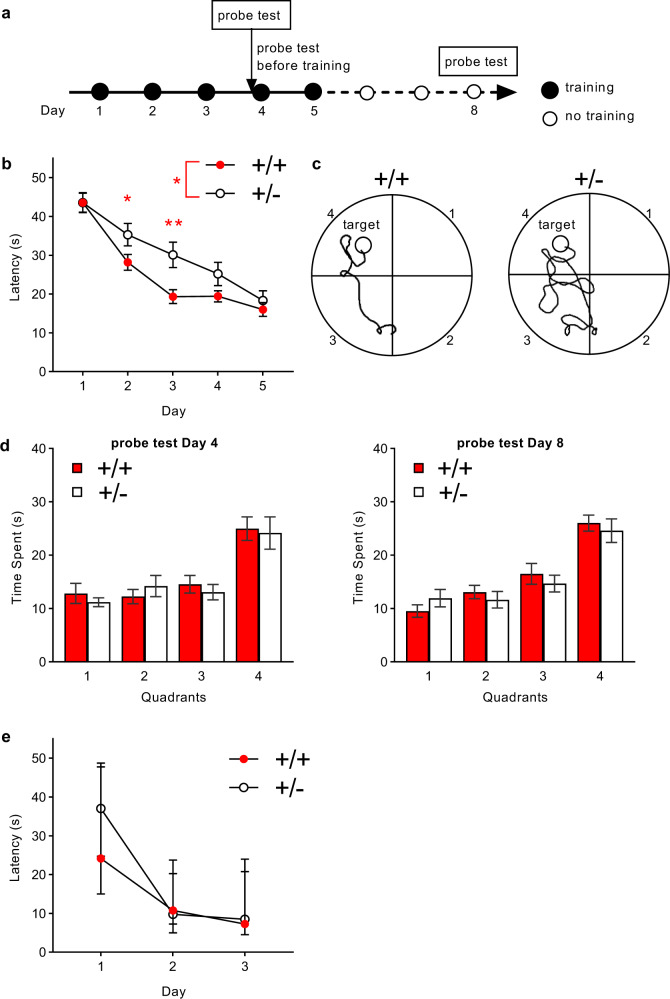
Performance in the Morris water maze test. **a** Experimental design. **b** The mean (± standard error of the mean [SEM]) escape latency in seconds (s) to the platform during acquisition is plotted against days. Compared with +/+ mice, +/− mice exhibited delayed acquisition (Genotype, F(1,26) = 4.643, *p* = 0.041, ∗[; Day, F(4,104) = 55.490, *p* < 0.001; Genotype × Day, F(4,104) = 2.329, *p* = 0.061). The overall genotype effect was primarily due to robust differences on Day 2 (*, *p* < 0.05) and Day 3 (**, *p* < 0.01), as determined by Newman–Keuls post hoc tests. +/+, *n* = 14; +/–, *n* = 14. **c** Representative swim paths of a +/+ mouse and +/− mouse on the third training day. The target quadrant included the hidden platform. **d** The mean (± SEM) time spent during recall probe tests before training on Day 4 (left) and Day 8 (right). Regardless of the quadrant, there were between-genotype differences on Day 4 (Genotype, F(1,26) = 5.597, *p* = 0.026; Quadrant, F(3,78) = 14.259, *p* < 0.001; Genotype × Quadrant, F(3,78) = 0.295, *p* = 0.829) and Day 8 (Genotype, F(1,26) = 10.207, *p* = 0.004; Quadrant, F(3,78) = 24.031, *p* < 0.001; Genotype × Quadrant, F(3,78) = 0.562, *p* = 0.642). The significant main effects of genotype on both days primarily resulted from the generally lower amounts of time spent in three out of the four quadrants in +/− mice (Day 4, Quadrants 1, 3, and 4; Day 8, Quadrants 2, 3, and 4). **e** The mean (± SEM) escape latency in the visible cue task. A separate set of mice underwent examination using this version of the Morris water maze. +/+ and +/− mice equally acquired this task (Genotype, F(1,17) = 1.861, *p* = 0.190; Day, F(2,34) = 52.313, *p* < 0.001; Genotype × Day, F(2,34) = 1.229, *p* = 0.305). +/+, *n* = 8; +/−, *n* = 11.

*Tbx1* +/− mice exhibited delayed spatial memory acquisition in the Morris water maze (Fig. 5a–c). In contrast, there was no between-genotype difference in the probe test (Fig. 5d) or during visual cue memory acquisition (Fig. 5e). These data indicate that *Tbx1* heterozygosity impairs the acquisition speed of fimbria-dependent spatial reference memory, but not its retention or recall, or fimbria-independent visual cued memory.

#### *Tbx1* heterozygosity slows the acquisition of discrimination and cognitive flexibility

Individuals with 22q11.2 hemizygous deletion also exhibit impairments in executive functions [11]. Congenic mouse models of 22q11.2 hemizygosity require an increased number of trials to reach the criteria for simple discrimination and reversal learning [58] or extradimensional shifting (EDS) [59]. However, the individual 22q11.2 genes contributing to impairments in executive functions remain unclear. In humans, prefrontal cortical lesions increase the number of trials required to reach the criterion of attentional set shifting; on the other hand, lesions of the hippocampus and its connections affect the latency for completing each trial [60]. In rodents, orbitofrontal cortical lesions increase the number of trials required to achieve reversal of the intradimensional set (IDS-IV rev) [61], but the rodent brain regions critical for the speed to complete each trial of attentional set shifting are not clear.

*Tbx1* +/− mice lacked detectable white matter alterations in the basal forebrain or cortex (see Fig. S2–S5) but exhibited altered myelination in the fimbria. Thus, we reasoned that *Tbx1* +/− mice may exhibit altered latency in completing attentional set shifting but may be unaffected in terms of the aspect of attentional set-shifting task requiring the prefrontal cortex (number of trials needed to reach a criterion).

**Fig. 6:**
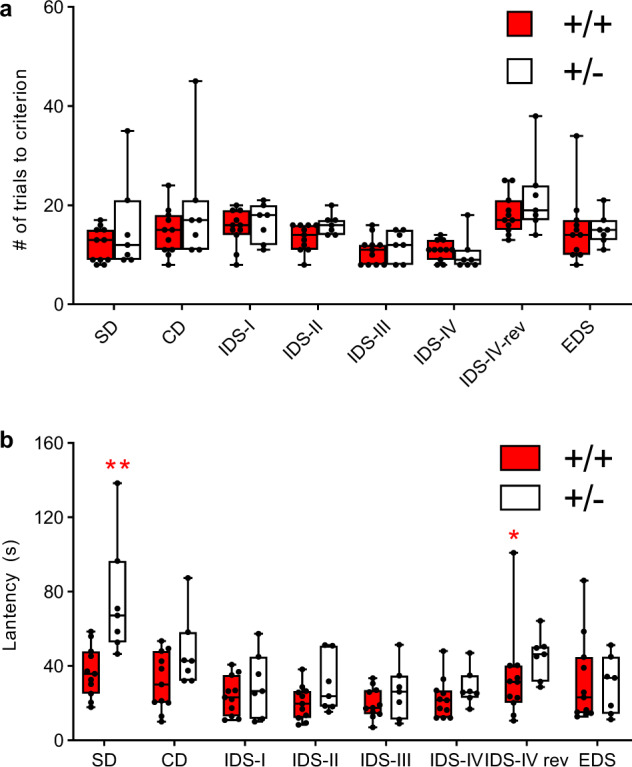
Attentional set shifting. Box-and-whisker plots of (**a**) the number of trials required to reach the criterion (i.e., eight consecutive correct choices) and (**b**) latency to complete each trial during the first five correct choices. Since the normality assumption was violated (**a**, *p* = 0.002, at IDS-IV of +/−; **b**, *p* = 0.001, at IDS-IV rev of +/+), we analyzed both sets of data using a generalized linear mixed model. There was no between-genotype difference in the number of trials taken to reach the criterion (Genotype, F(1,16) = 1.965, *p* = 0.180; Genotype × Phase, F(7,112) = 0.824, *p* = 0.569). SD simple discrimination, CD compound discrimination, IDS intradimensional shift, rev reversal, EDS extradimensional shift. +/− mice were consistently slow in completing this task in a phase-dependent manner (Genotype, F(1,16) = 10.010, *p* = 0.006; Genotype × Phase, F(7, 112) = 2.566, *p* = 0.017). Mann–Whitney nonparametric post hoc comparisons revealed a significant between-genotype difference in latency to completing the two phases of SD (**, *p* < 0.01) and IDS-IV rev (*, *p* < 0.05). +/+ = 11, +/− = 7.

There was no between-genotype difference in the number of trials required to complete each phase of attentional set shifting (Fig. 6a). In contrast, +/− mice were slower in completing each trial of attentional set shifting, most significantly in simple discrimination (SD) and IDS-IV rev (Fig. 6b).

#### *Tbx1* heterozygosity has no detectable effects on olfactory responses

Consistent with the lack of detectable alterations in the white matter integrity of the neocortex, amygdala, and olfactory bulb (Fig. S2–S5), there was no between-genotype difference in responses or habituation to nonsocial and social olfactory cues (Fig. S10). This observation suggests that *Tbx1* heterozygosity does not exert nonspecific effects on visual or olfactory perception or on the general motivation to approach an object or odorants.

In sum, our behavioral analysis identified a highly demarcated deficit in the acquisition speed of fimbria-dependent cognitive tasks in *Tbx1* +/− mice.

## Discussion

The cellular, structural, or cognitive consequences of loss-of-function *TBX1* variants in humans remain unclear. Our parallel analysis of structural and behavioral measures in a congenic mouse model of *Tbx1* heterozygosity indicated that *Tbx1* deficiency caused highly demarcated changes in the structural features of the brain, including reduced production of oligodendrocytes, suboptimal composition of myelination in the fimbria and corpus callosum, and loss of large myelinated axons in the fimbria. These structural alterations impacted fimbria-dependent cognitive functions: *Tbx1* heterozygous mice exhibited increased latency to acquire spatial memory and simple discrimination and reversal of intradimensional shift. These effects of *Tbx1* heterozygosity on structures and functions are likely due, at least in part, to its enriched expression in adult progenitor/stem cells in the mouse brain [26]. Our single-gene analysis provides a valid first step for deconstruction and reconstruction of the mechanistic composition of CNV-encoded genes in terms of their association with specific behavioral and structural dimensions. As individuals with 22q11.2 hemizygosity exhibit impairments in cognitive speed [11, 13, 62] as well as altered white matter integrity in the hippocampal-projection fibers [16–18, 63], our analyses of a single 22q11.2-encoded gene offer insight into the genetic and cellular substrates of these structural and behavioral alterations in carriers of 22q11.2 hemizygosity. There are inherent limitations in assessing the cognitive dimensions in mouse models due to species-specific differences between mice and humans. The ultimate validation of our mouse data would be achieved by testing therapeutic interventions based on our hypothetical mechanisms underlying defective cognitive speed in humans.

The absence of large myelinated axons in the fimbria of +/− mice may be attributable to a reduced number of oligodendrocytes. Our in vivo data indicated that *Tbx1* heterozygosity impacts *Ng2*, a molecule required for the production of oligodendrocyte precursor cells, in the fimbria. Our in vitro analysis further revealed that fewer oligodendrocytes are produced from postnatal progenitor cells derived from +/− mice. There are several possible reasons for the selective loss of large myelinated axons in the fimbria. Given their higher need for metabolic support from myelin and oligodendrocytes [64–66], large axons may degenerate. Alternatively, but not mutually exclusive, *Tbx1* heterozygosity may lead to selective inactivation of large-diameter axons and consequently reduced myelination of those axons, as oligodendrocytes tend to myelinate electrically active axons [67]. In either case, the remaining oligodendrocytes may have instead myelinated medium axons in the fimbria, which would explain the hypermyelination of medium axons observed in the present study. While little is known about the functional contribution of large myelinated axons in the fimbria to cognitive functions, our data provide a potential cellular basis for cognitive speed for further investigation.

The observations that *Ng2* mRNA was reduced in the fimbria (see Fig. 3a), but markers of mature oligodendrocytes were not (see Fig. 3a), are seemingly difficult to reconcile. Given that the fimbria of +/- mice contained hypermyelinated medium axons but was devoid of large myelinated axons, it is possible that the effects of these positive and negative alterations on the net amount of MBP and MOG mRNA cancel out in the fimbria. Additionally, as myelin was selectively reduced in the anterior fimbria only (see Fig. S7ab), such a regionally limited effect may be difficult to detect in the whole fimbria tissue used for qRT-PCR.

It is striking that, among all the brain regions scanned, the fimbria was the only region that showed a statistically significant reduction in FA values in *Tbx1* +/− mice. The reason for this selectivity remains unclear, but may be due to the postnatal onset of myelination in the rodent brain [40] and highly limited expression of Tbx1 protein in the subventricular zone and granule cell layer of the hippocampus in the postnatal mouse brain [26]. Oligodendrocytes in the subventricular zone postnatally migrate to the fimbria and corpus callosum [50]. Reduced myelination of medium-diameter axons in the corpus callosum—as well as a lack of large myelinated axons in the fimbria—may have also occurred due to reduced postnatal migration of oligodendrocytes from the subventricular zone of *Tbx1* +/− mice. More work is needed to determine how local oligodendrocyte precursor cells in the fimbria and oligodendrocytes postnatally provided from the subventricular zone contribute to the myelination of axons of different sizes.

The fornix is an extension of the fimbria and shows one of the largest reductions in FA values among all the brain regions in carriers of 22q11.2 hemizygous deletion [17, 18]. The reduction in FA is more severe in patients with psychosis than without psychosis [17]. FA reductions are larger in idiopathic cases of schizophrenia than in bipolar disorder, major depression, PTSD, or OCD [16–18]. The fimbria carries efferent fibers, via the fornix, to the anterior thalamic nuclei, mammillary body, nucleus accumbens, lateral septum, and afferents from the medial septum in rodents [68]. Our work offers a basis for future studies to delineate the precise axons, targets, and neuronal types underlying slow cognitive speed.

A previous study reported that acquisition speed, retention, and recall of spatial reference memory in the Morris water maze were normal in a mouse model of 22q11.2 hemizygosity [69]. It seems inconsistent that the deletion of *Tbx1*, one of the 22q11.2 genes, slowed the acquisition speed of spatial memory in the Morris water maze, but a mouse model of 22q11.2 deletion, including *Tbx1*, did not. There are several potential reasons for this. First, many mouse models of CNVs have failed to recapitulate behavioral deficits, while models of single CNV genes exhibit robust phenotypes [31, 70, 71]. Why mouse models of the entire 22q11.2 hemizygous deletion are normal in many cognitive tasks remains unclear. Second, unlike the 22q11.2-deletion mouse model used by Drew et al., our *Tbx1* +/− mice were fully congenic with homogeneous genetic backgrounds in +/+ and +/−. Substantial allelic differences are expected between the control and mutant non-congenic mice near the deletion region in noncongenic mice, which could potentially contribute to the apparent absence of phenotypic differences [70]. Third, in the study of Drew et al., the same mice were first trained and tested in the visual cue version and subsequently in the spatial memory version without any interval. The prior training in the visual task might have ameliorated the potential deficit in subsequent testing in the hidden-platform version [72] In contrast, we used different sets of mice for the visual and spatial memory versions of the Morris water maze to avoid a carryover effect of the prior training on subsequent performance.

The structural alterations and cognitive deficits observed in the present study are not unique to *Tbx1* heterozygosity or 22q11.2 CNVs. Lower FA values have been reported in the fimbria/fornix of individuals with idiopathic ASD [73] and schizophrenia [18, 74]. Slow processing speed in individuals with idiopathic ASD is correlated with low FA values, but not with MD, RD, or AD values, in the whole brain [75]. Individuals with idiopathic ASD also exhibit impairments in difficult cognitive tasks [76]. A selective loss of extralarge myelinated axons has been observed in the brains of humans with ASD [77]. Moreover, patients with idiopathic schizophrenia exhibit impaired processing speed across numerous cognitive dimensions, including attention, memory, spatial processing, emotional identification, and sensorimotor capacity [78, 79]. Previous studies have also reported that other oligodendrocyte-related genes are dysregulated in brain samples from individuals with ASD and genetic mouse models for ASD [80–84]. Taken together, our findings open a new window for investigating the potential substrates of altered cognitive speed in carriers of *TBX1* SNVs, 22q11.2, and other CNVs, and in idiopathic cases of ASD and schizophrenia.

## Supplementary information

Supplementary Information

Supplementary Figure 1

Supplementary Figure 2

Supplementary Figure 3

Supplementary Figure 4

Supplementary Figure 5

Supplementary Figure 6

Supplementary Figure 7

Supplementary Figure 8

Supplementary Figure 9

Supplementary Figure 10

## Data Availability

All data are available upon request. Mice are available through the Material Transfer Agreement.
