# Supplementary material for: Tbx1, a gene encoded in 22q11.2 copy number variant, is a link between alterations in fimbria myelination and cognitive speed in mice
Source: Mol Psychiatry. 2021 Nov 5;27(2):929–38. doi: 10.1038/s41380-021-01318-4 (PMC9054676; doi:10.1038/s41380-021-01318-4)
Supplement: Supplementary file 1 — Supplementary Information [file 41380_2021_1318_MOESM1_ESM.docx]

Supplementary Materials for

*Tbx1*, a 22q11.2-encoded gene, is a link between alterations in fimbria myelination and cognitive speed in mice

Takeshi Hiramoto^1†^; Akira Sumiyoshi^2,3†^; Takahira Yamauchi^1†^; Kenji Tanigaki^5^; Qian Shi^6^, Gina Kang^1^; Rie Ryoke^2^; Hiroi Nonaka^2^; Shingo Enomoto^7^; Takeshi Izumi^8^; Manzoor A. Bhat^6^; Ryuta Kawashima^2^; Noboru Hiroi^1,6,10*^

*****Corresponding author: Noboru Hiroi, PhD, hiroi@uthscsa.edu

**This PDF file includes:**

Methods

Tables S1 to S5

Reference

**METHODS**

**Mice**

The protocols for animal handling and use were approved by the Animal Care and Use Committee of the Albert Einstein College of Medicine, University of Texas Health Science Center at San Antonio and Tohoku University in accordance with National Institutes of Health (NIH) guidelines.

*Tbx1*^+/-^ mice This mouse model was a congenic strain with a C57BL/6J background. The original non-congenic *Tbx1*^+/-^ mouse was backcrossed onto C57BL/6J inbred mice for >10 generations to control for biased genetic backgrounds ^1^. Given that there are no sex biases in the prevalence of schizophrenia or ASD ^2^, set-shifting, spatial working memory, spatial planning, processing speed, or other cognitive domains ^3, 4^ among carriers of 22q11.2 hemizygosity, we used either male or female mice for the various analyses.

We determined genotypes of mice using three primers: forward TTGGTGACGATCATCTCGGT and reverse ATGATCTCCGCCGTGTCTAG to detect the +/+ genotype, as well as an additional reverse AGGTCCCTCGAAGAGGTTCA to detect the +/- genotype.

The experimenter was blinded to genotypes for both anatomical and behavioral analyses.

**DTI-MRI**

We performed *ex vivo* MR scanning to achieve a high resolution and high signal-to-noise ratio since it allows a long scan time and involves the use of a contrast agent. In accordance with standard procedures ^5^, 4-month old female mice were anesthetized using pentobarbital (60 mg/kg, i.p., Nancalai Tesque, Kyoto, Japan, cat#02095-04) and transcardially perfused using 30 mL of 0.01 M phosphate-buffered saline (PBS, Nancalai Tesque, Kyoto, Japan, cat#14249-95) that contained 2 mM of ProHance (Bracco-Eisai Co., Ltd, Tokyo, Japan, cat# 22100AMX00462000) and 1 μL/mL heparin (1,000 USP units/mL, Nancalai Tesque, Kyoto, Japan, Cat# 17513-96), followed by 30 mL of 4% paraformaldehyde (PFA; Wako, Tokyo, Japan, Cat# 161-20141) containing 2 mM ProHance. The head was decapitated, following which the skin, lower jaw, ears, and cartilaginous nose tip were removed. The skull structure containing the brain tissue was post-fixed in fixative (4% PFA and 2 mM ProHance) overnight at 4°C. Subsequently, it was transferred to buffer (0.01 M PBS, 0.02% sodium azide (Wako, Tokyo, Japan, Cat#195-11092), and 2 mM ProHance) at 4°C overnight. Next, the brain tissues were placed in fresh buffer (0.01 M PBS, 0.02% sodium azide + 2 mM ProHance). Immediately before scanning, we immersed *ex vivo* mouse brains in Fomblin (Sigma-Aldrich, St Louis, MO, Cat#317926), which is a perfluorocarbon that reduces susceptibility artifacts at the interface and limits intra-scanning sample dehydration.

MRI data were acquired using a 7.0-T PharmaScan 70/16 system with a 23-mm diameter birdcage Tx/Rx coil specifically designed for the mouse brain (Bruker Biospin, Ettlingen, Germany) using standard operational software (Paravision 6.0.1). We acquired triplot images to ensure proper sample positioning with respect to the magnet isocenter. Shim gradients were adjusted using the MAPSHIM protocol with an ellipsoid reference volume covering the whole brain. We obtained diffusion-weighted images using a standard spin-echo 2D pulse sequence using the following parameters: repetition time = 4,158 ms, echo time = 42 ms, field of view = 15 × 12 mm^2^, matrix size = 100 × 80, in-plane resolution = 0.15 × 0.15 mm^2^, number of slices = 50, slice thickness = 0.3 mm, diffusion gradient duration = 6 ms, diffusion gradient separation = 30 ms, b-value = 2,000 s/mm^2^, number of diffusion directions = 30, number of b0 images = 1, effective spectral bandwidth = 30 kHz, fat suppression = on, and number of averages = 10. The diffusion-weighted images was acquired at 22^0^C-26^0^C for 22 h per mouse.

Acquired images were processed using the Advanced Normalization Tools ([http://stnava.github.io/ANTs/](http://stnava.github.io/ANTs/)according)) and FMRIB Software Library (FSL) software packages (<https://fsl.fmrib.ox.ac.uk/fsl/fslwiki>). The procedure for image processing was as follows: (i) Image reconstruction was performed using Paravision software and converted to the NIfTI format using “DSI Studio” software (<http://dsi-studio.labsolver.org/>); (ii) eddy-current induced distortions were corrected using the eddy_correct tool of FSL; (iii) individual reference b0 images were manually skull-stripped using ITK-SNAP software ([http://www.itksnap.org](http://www.itksnap.org/)); (iv) other subject b0 images were registered to the reference image and skull-stripped; (v) scalar images were reconstructed using the DTIFIT tool of FSL; (vi) b0 and scalar images were manually rotated and translated to ensure that the coordinate origins occupied the anterior commissure midpoint to roughly match the standard reference space; (vii) b0 and scalar images were resampled onto an 0.15-mm isotropic voxel; (viii) the Minimum Deformation Template (MDT) space was constructed using all subject b0 and scalar images, including FA, AD, MD, and RD images; (ix) b0 and scalar images were warped to the MDT space; (x) the mean b0 image was computed, manually skull-stripped, and registered to the atlas image; and (xi) the mean FA, AD, MD, and RD values in each structure were computed and statistically analyzed.

**Black-Gold II staining**

Two-to three-month-old female mice were perfused with saline and 4% paraformaldehyde as per the standard protocol ^6^. We mounted a pair of free-floating 40-μm thick coronal sections from a +/+ mouse and a +/- littermate as the upper and lower rows, respectively, on the same slides (3-4 section pairs per slide) to control for cross-slide staining variations. Care was taken to mount a section pair with similar coordinates from +/+ and +/- mice on the upper and lower rows of a slide.

The degree of myelination was examined using Black-Gold II staining ^7^. Black-Gold II is an aurohalophosphate complex that directly stains myelin within the CNS. Black-Gold II and sodium thiosulfate solution (AG105, Millipore, Temecula, CA) were heated to 60°C. Slide-mounted sections were rehydrated in filtered water, transferred to pre-warmed Black-Gold II solution, and incubated at 60°C for >12 min. Subsequently, the sections were rinsed in filtered water twice for 2 min each, transferred to sodium thiosulfate solution, and incubated for 3 min at 60°C. Finally, the sections were rinsed three times in filtered water for 2 min each and cover-slipped.

We semi-quantified gold-staining within the fimbria and corpus callosum using a Keyence microscope and its controller (BZ-X810 and BZ-X800E, Keyence, Austin, Texas). Under a light microscope, staining blocked light penetration through the sections and registered as less bright. This property was employed for semi-quantitative analysis.

We focused on the fimbria (Bregma ~-0.82 to ~-1.94mm), as RT-MRI suggested that +/+ and +/- mice most differed in FA values in this region. The fimbria and corpus callosum regions were delineated as targets (see **Fig. S6**). As the fimbria has distinct shapes along the antero-posterior axis, we further divided the anterior fimbria into two parts: anterior subregion (Bregma ~-0.82 to ~–1.33 mm) and posterior subregion (~-1.34 to -1.94mm). We also measured the staining intensity of the anterior two thirds (Bregma ~-0.82 to ~–1.33 mm) and posterior one third (Bregma ~-1.34 to -1.94mm) of the isthmus of the corpus callosum.

The Keyence software yields brightness (B) values as integration values within a range of threshold values from 0 to 255. The threshold value acts as a filter and determines the level of light that is allowed to penetrate through a section. We observed that sampling pixels gradually saturated areas where tissues exist up to a threshold unit value of 137, above which pixels started to appear non-specifically in areas devoid of tissue (e.g., blood vessels and between-tissue gaps). Thus, signals are maximally detected without false positive signals at this threshold value. This threshold was consistently used in the analysis of staining signals. Since B represents the sum of all integration values within the delineated area, it is affected by the size of the area. Because the target area size varied from section to section, we computed B per area (A) unit of the target (t) region (i.e., tB/A) in the fimbria and corpus callosum (**Table S2, Step 1**).

Although we minimized slide-to-slide variations in staining intensity by dipping a set of slides in the same Black-Gold II solution, there was still variation. This was observed as varying non-specific baseline staining across sections. To correct for this variation, we adjusted the tB/A value based on the degree of non-specific staining. We chose a 250 μm × 250 μm cortical area above the target fimbria and corpus callosum, where gold-labeling was negligible. We defined it as a negative control (nc) area where B/A values represent non-specific staining (**Table S2, Step 2**). The threshold unit for genuine tissue signals was 255 in the cortex, above which signals started to appear in areas with no tissue. We next chose a section with the maximum negative control B/A (max ncB/A) value and converted all ncB/A values to ratios (R=(max ncB/A)/(ncB/A), **Table S2, Step 3**).

Next, the tB/A value was multiplied by the R value, such that an under-estimated brightness signal due to non-specific staining (i.e., low B/A value) was rectified proportionally to the relative degree of non-specific staining (tB/A^adj^=(tB/A)*R, **Table S2, Step 4**).

As staining intensity is inversely proportional to the tB/A^adj^ value, greater gold staining indicates that less light penetrates a section. The inverse value of tB/A^adj^ was calculated (1/(tB/A^adj^), **Table S2, Step 5**) and multiplied by 10^2^ (corpus callosum) or 10^3^ (fimbria) to express at least one natural number before the decimal point.

**EM analyses**

Two- to three-month-old male mice were anesthetized using 4-5% isoflurane in a chamber, and anesthesia was maintained with 2.0-3.5% isoflurane using a nose-cone vaporizer. The animals were intracardially perfused with 100 mL of 0.9% physiological saline followed by approximately 250 mL of freshly prepared 0.1 M sodium cacodylate buffer (pH 7.4; Electron Microscopy Sciences, Hatfield, PA, cat #11653), which contained 2.5% glutaraldehyde (Electron Microscopy Sciences cat #16320) and 2.5% PFA (Electron Microscopy Sciences cat #19202). Next, the brains were split into two hemispheres and post-fixed in fixative at 4°C for 2 weeks. Samples from the target areas (fimbria and corpus callosum) were obtained using a vibratome and placed in 0.1 M sodium cacodylate buffer overnight. The tissues were then rinsed three times for 10 min each in 0.1 M cacodylate buffer to remove aldehydes, following which they were placed in a mixture (500 μL) of 2% OsO_4_ (Electron Microscopy Sciences, cat#19150) and 0.1 M sodium cacodylate buffer for 1 h. The tissue samples were agitated and shaken, rinsed (3 x 5 min 0.1 M Na cacodylate), and dehydrated twice in a series of ice-cold ethanol solutions for 5 min each (30% ethanol; 50% ethanol; 70% ethanol; 90% ethanol; 95% ethanol) and three times in 100% ethanol for 10 min. Next, the tissues were rinsed twice in propylene oxide for 30 min each (Polysciences, Inc., Warrington, PA, cat# 00236–1). This was followed by incubation on a mixer at room temperature overnight in an approximately 1 mL mixture of 1 part propylene oxide and 1 part Polybed resin solution (Poly/Bed® 812 Embedding Media, Polysciences, Inc., cat# 08791–500; Dodecenylsuccinic anhydride (DDSA, Polysciences Inc., cat# 00563–450), nadic methyl anhydride (NMA, Polysciences Inc., cat# 00886–500), and 2,4,6-Tris-(dimethylaminomethyl)phenol (DMP-30, Polysciences, Inc., cat# 00553–100). On the next day, the Polybed resin/propylene oxide solution was removed, and the tissues were incubated for 24 h in 100% Polybed solution on a mixer at room temperature. Tissues were removed from the Polybed resin and placed in a mold, following which fresh polyresin was added. After the resulting bubbles had disappeared, the tissues in the mold were incubated at 55°C for 36 h. Subsequently, they were processed at the Electron Microscopy Laboratory of the UT Health Science Center in San Antonio using the in-house procedure ^8^. The tissues were cut at 1 µm and stained using 0.1% toluidine blue/0, 0.1% methylene blue/0, and 0.1% azure II in 1% sodium borate buffer. Next, 100-nm thick sections were cut and collected on 300 hexagonal mesh copper grids (Electron Microscopy Sciences, cat # T300H-Cu). In each set of five grids, three were stained, and two were left unstained. Staining was performed using 5% uranyl acetate in 50% methanol and Reynold’s lead citrate ^9^. We measured the diameters of myelinated axons and their axon portions.

**qRT-PCR**

We used 2-3 month-old female *Tbx1* +/+ and +/- littermates. We dissected the fimbria and corpus callosum based on the shape and landmark of each region. The fimbria's whole dorsoventral and mediolateral extents from Bregma -0.46 mm to -0.82 mm were taken. The medial + 0.5 mm was excluded because it contains the triangular septal nucleus. The whole dorsoventral extent of the corpus callosum (Bregma from -0.46 mm to -0.82 mm; ML, +1.0 mm) was taken. Care was taken to exclude the cingulum. However, we do not rule out the possibility that the triangular septal nucleus and cingulum were included in the samples.

Total RNA was extracted from brain regions of adult mice using an RNeasy Plus Mini Kit (Cat#74134, Qiagen, Germantown, USA), in accordance with the manufacturer’s instructions. cDNA was synthesized from total RNA using SuperScript IV VILO master mix (Cat# 11766050, Invitrogen, Carlsbad, USA). Quantitative PCR reactions were performed in triplicate on QuantStudio 6 Flex Real-Time PCR Systems (Cat#4485694, Applied Biosystems, Waltham, USA) using the TaqMan Fast Advanced Master Mix (Cat#4444963, Applied Biosystems, Waltham, USA). The Taqman probes are listed in the Supplementary Material (**Table S3**). Data were analyzed using the ΔΔCt method and normalized to the reference gene Cyc1.

***In vitro* analysis of oligodendrocytes**

We used P21 +/+ (n =9) and +/- mice (n = 5) chosen from five litters. Progenitor cells were isolated from the lateral ventricular walls of both hemispheres. Two 1mm slices were taken from each of both hemispheres, and tissues that include the subventricular zone were dissected. Each culture was prepared using tissue from a single mouse. The tissues were dissociated using a Neural Tissue Dissociation Kit (P) (130-092-628, Miltenyi Biotech GmbH, Germany). We did not purify neural progenitor cells with antibodies; thus, our cells contained different types of proliferating progenitor cells that generate neurons and oligodendrocytes. The cells were cultured in a medium (DMEM/F12 [11320–033, Gibco, CA, USA]) supplemented with N2 (17502048, Gibco), B27 (17504044, Gibco), epidermal growth factor (EGF) (20 ng/ml) (AF100-15, Peprotech, Cranbury, NJ, USA), and fibroblast growth factor 2 (FGF2) (10 ng/ml) (100-10B, Peprotech). After two to three passages, the cells were dissociated from the spheres and seeded on a Matrigel (356234, BD Biosciences, Bedford MA, USA)-coated slide chamber (154534, Nunc, Rochester, NY, USA). To promote differentiation, the cells were cultured for 4 days in medium supplemented with 5% fetal calf serum. Next, the cells were fixed using 4% PFA for 15 min and processed for immunofluorescence staining, using a purified mouse monoclonal O-4 antibody (1:50, MAB345, Millipore, Burlington, MA, USA) for 12 h at 4°C after blocking for 30 min at room temperature with 5% donkey serum (S30-100ml, Millipore, MA, USA). Subsequently, the cells were incubated for 30 min at room temperature with Goat anti-Mouse IgM (Heavy Chain) Secondary Antibody, Alexa Fluor 647 (1:1000, A21238, Molecular Probes, OR USA). Nucleus staining was performed using 4',6-diamidino-2-phenylindole (DAPI) (3 mM, D3571, Molecular Probes). Cells were counted from four randomly selected fields per culture under a confocal microscope (TCS SP8, Leica, Germany), and the average score was obtained.

**Behavioral analysis**

The mice were randomly assigned to experimental groups and tested during the light phase between 10 AM and 5 PM.

# Morris water maze. Separate groups of 2-month-old male mice were used for the hidden and visible platform versions of the Morris water maze test. The water tank (103 cm in diameter; ~914 lx) contained white Prang® (Dixon Ticonderoga® Appleton, WI, Model#21609) Ready-to-Use Paint (Item #: 738062, Model #: 21609/21949, Staples, New York, NY) mixed in water (24 ± 2°C). A circular platform (10 cm in diameter) was submerged 1 cm below the surface in the middle of one quadrant. Cues were placed on the wall 40 cm from the tank edge. The water was changed after testing on days 3 and 5.

The hidden platform version involved 10 sessions conducted over 5 days (two daily sessions at intervals of 2­–4 hours). Each session included four 60-s trials conducted at 15-min intervals. The platform location remained constant (Quadrant 4); however, the entry points were semi-randomly changed across the trials. Before the fourth day of the hidden platform training, we performed a 60-s probe trial for which the platform was removed. The entry point for the probe trials was the quadrant opposite to the target quadrant. An additional probe trial was conducted 72 h after the fifth day of hidden-platform training.

The cued platform version involved six sessions conducted over 3 days (two daily sessions at intervals of 2–4 h with each session having two 60-s trials at 15-min intervals). The platform was marked using a flag placed above the water surface and visible to the mice. The platform locations were randomly assigned to each trial. The mice were placed in the maze from four equally spaced points along the pool perimeter, and the entry-point sequence was randomly chosen. For each placement, the animals were placed facing the sidewalls. The sequence of four start positions (north, south, east, and west) varied across the trials.

In both the hidden and cued platform versions, all animals were allowed to remain on the platform for 30 s. In case they did not reach the platform during the 60-s test, the experimenter placed and left the animal on the platform for 30 s. During the 15-min inter-trial interval, the mouse was dried using a paper towel and placed in an empty cage stuffed with a dry paper towel.

Attentional set shifting: This test was performed using a procedure optimized for mice ^10^, with a slight modification. Two- to three-month-old male mice were individually housed and food-deprived to reduce the bodyweight to 85% of the *ad libitum*feeding weight, and this bodyweight was maintained throughout the testing period.

The mice were taken to the test room 1 h before the start of the training session. A single bowl containing 1/2 of a Honey Nut Cheerio buried in one medium stimulus sprinkled with an odor stimulus was placed in the home cage. This training used all possible combinations of exemplars of both dimensions (i.e., odor and medium) for subsequent use in the eight phases of attentional set shifting. The mice completed four daily trials, each involving a unique combination of medium and odor stimuli. The bowl was immediately removed from the home cage after the mouse had dug up the food pellet and eaten it. Each trial lasted approximately 1–2 min. After completing the daily training trials, the mouse was placed in a new home cage with fresh bedding.

Next, we conducted a one-day habituation session in the attentional set-shifting apparatus (outer dimensions: height (H), 15 cm x width (W), 19.2 cm x length (L), 49.2 cm; inner dimensions: H, 14.4 cm x W, 18.3 cm x L, 48.3 cm; ~914 lux). The apparatus was divided into two goal compartments (W, 9 cm x L, 14 cm each) and one start compartment (W, 18.3 cm x L, 33.9 cm) using 4.8-mm-thick walls. The mouse explored the apparatus arena, which included a plastic weigh boat containing water in the start compartment. The two goal compartments lacked bowls. After 3 minutes, the partition door was placed to confine the mouse to the start compartment. After another 3 minutes, the door was removed to allow the mouse to freely explore all three compartments. The two 3-min sessions were repeated five times.

On the next day, training began in the attentional set-shifting session apparatus. The water tray remained in the starting compartment during testing and initial re-training. The two bowls in the goal compartments contained two medium stimuli (e.g., alpha dri and paper chips) without odor stimuli; moreover, they were both baited using food. Both media were used for the subsequent SD sessions. The partition door was placed to confine the mouse to the start compartment. Care was taken to remove the door when the mouse was not sniffing or facing it. Initially, the mice underwent four re-training trials to retrieve the food from the bowl. The positions of both medium-containing bowls were randomized in each trial. We placed an eighth of a Honey Nut Cheerio on top of (trial 1), half-buried within (trial 2), slightly covered by (trial 3), and completely buried within (trial 4) the media. Each trial ended when the mouse had eaten food from both bowls.

Subsequently, the mice underwent a series of discrimination tests. Initially, each mouse was placed in the start compartment with a partition door. The two goal compartments contained one baited bowl (an eighth of a Cheerio piece completely buried in the medium) and one un-baited bowl; moreover, the position of the baited bowl was randomized across the trials. The partition door was then lifted. Each trial ended when the mouse had made a correct choice and had eaten the reward. If the mouse dug into the un-baited bowl, it was removed after the mouse had spontaneously left the un-baited compartment. A time-out was given if the mouse did not dig in any bowl for 3 min, which involved removal of the bowl from the test arena and subsequent resumption of the trial using a different medium/odor pair. In case of three consecutive time-outs, the testing was ended and resumed the next day. Each of the eight phases ended when the mouse had made eight consecutive correct choices or after 50 trials per day, whichever came first. If a mouse made eight consecutive correct choices within 50 trials, a new phase was administered the next day. After each test trial and when changing mice, the arena and bowls were wiped using 70% ethanol.

The attentional set-shifting phases were as follows (**Tables S4** and **S5**). For the SD phase, there were two choices for the two relevant dimensions. The compound discrimination (CD) phase was similar to the SD phase, except that a new correct compound (O1&M1 and O1&M2) was added. The IDS IV phase involved CD using two novel exemplars from relevant and irrelevant dimensions for each IDS with the same relevance. The IDS IV rev phase involved the same exemplar set as the IDS IV phase, except that the correct choice within the relevant dimension was reversed. The extradimensional shifting (EDS) phase involved novel CD, except that the correct choice was an exemplar of the previously irrelevant dimension up to IDS-IV rev. The order of discrimination and exemplars was similar for all mice. The exemplar choice and correct bowl position were pre-determined using a random number table.

The standard mouse SD procedure uses O1 plus M1 and O2 plus M1 ^10^. Our modified SD procedure used a combination of two dimensions (O1 plus M1 as the correct discriminants and O2 plus M2 as the incorrect discriminants). Our pilot study indicated that, compared with +/+ mice, +/- mice exhibited a longer latency to complete this modified task.

We determined the number of trials taken to reach eight consecutive correct choices and the latency to complete a trial from the trial start to the time point when the mice began eating the food pellet.

Olfactory responses to social and non-social cues. This test was conducted in a test cage (L, 28.5 cm × W, 17.5 cm × H, 12.5 cm) that had been divided into a 19.5 cm-long compartment and a 9 cm-long compartment using a partition wall with a 5 cm (H) × 5 cm (W) opening; ~430 lux. The test was conducted as previously described ^11^, with slight modifications. First, 2-month-old male mice were habituated to the apparatus for 15 min. A filter paper scented with a test odor was placed in a 1-ml Eppendorf tube containing small holes in the cap. Odors were sequentially tested as follows: water, almond, banana, urine from one non-littermate C57BL/6J male (NL1), urine from another non-littermate C57BL/6J male (NL2), urine from the first C57BL/6N mouse (NL1), urine from a non-littermate male +/- mouse (HT), urine from the dam (rm), urine from another litter’s mother (am), and urine from a non-littermate virgin female C57BL/6J mouse (v). We measured sniffing of the tube containing odorant-soaked filter paper during the 2-min trials. The mice underwent three 2-min trials for each odorant with an inter-trial interval of approximately 10 s; moreover, there was a 10-s interval between the three-trial session of one odorant and that of another. Urine was collected before testing and frozen at –20 °C until the test day. An Eppendorf tube with seven holes (one in the middle and six surrounding) in the cap was used for each trial. The tube was attached to the cage wall using Velcro. The filter paper (Whatman, #3698-325, Maidstone, England) was soaked in 10 µl of each odorant. During habituation, we placed dry filter paper in the tube.

**Statistical analysis**

We used GraphPad Prism 8.3.0 (GraphPad Software, San Diego, CA) and
IBM SPSS Statistics 26.0.0.0, IBM, Armonk, NY). Among-group and between-group comparisons of the data were performed using analyses of variance and Student’s two-tailed t-test, respectively. Normality and variance homogeneity of the data were evaluated using the Shapiro-Wilk test and Levene’s homogeneity of variance test, respectively. In case either assumption was violated, data were analyzed using a generalized linear mixed model, Kruskal Wallis tests, Mann-Whitney U-tests, and Wilcoxon non-parametric tests. The number of cases was analyzed using the χ^2^ test. Pearson’s correlation coefficients were compared by Fisher’s *r*-to-Z transformation. The minimum significance level was set at 5%. In case multiple tests were applied for a data set, the significance level was adjusted using the Benjamini-Hochberg correction, with a false discovery rate of 5%.

**Data availability**

All data that support the findings and conclusions are provided within the article and its supplementary information. All raw data and additional information will be made available upon request.

References:

1. Hiroi N. Critical Reappraisal of Mechanistic Links of Copy Number Variants to Dimensional Constructs of Neuropsychiatric Disorders in Mouse Models. *Psychiatry and Clinical Neurosciences* 2018; **72**(5)**:** 301-321.

2. Schneider M, Debbane M, Bassett AS, Chow EW, Fung WL, van den Bree MB *et al.* Psychiatric Disorders From Childhood to Adulthood in 22q11.2 Deletion Syndrome: Results From the International Consortium on Brain and Behavior in 22q11.2 Deletion Syndrome. *Am J Psychiatry* 2014; **171**(6)**:** 627-639.

3. Chawner S, Owen MJ, Holmans P, Raymond FL, Skuse D, Hall J *et al.* Genotype-phenotype associations in children with copy number variants associated with high neuropsychiatric risk in the UK (IMAGINE-ID): a case-control cohort study. *Lancet Psychiatry* 2019; **6**(6)**:** 493-505.

4. Gur RE, Yi JJ, Donald-McGinn DM, Tang SX, Calkins ME, Whinna D *et al.* Neurocognitive development in 22q11.2 deletion syndrome: comparison with youth having developmental delay and medical comorbidities. *Mol Psychiatry* 2014; **19**(11)**:** 1205-1211.

5. Dazai J, Spring S, Cahill LS, Henkelman RM. Multiple-mouse neuroanatomical magnetic resonance imaging. *J Vis Exp* 2011; (48)**:** e2497.

6. Boku S, Izumi T, Abe S, Takahashi T, Nishi A, Nomaru H *et al.* Copy number elevation of 22q11.2 genes arrests the developmental maturation of working memory capacity and adult neurogenesis. *Molecular Psychiatry* 2018; **23**(4)**:** 985-992.

7. Schmued LC. A rapid, sensitive histochemical stain for myelin in frozen brain sections. *J Histochem Cytochem* 1990; **38**(5)**:** 717-720.

8. Venable JH, Coggeshall R. A Simplified Lead Citrate Stain for Use in Electron Microscopy. *J Cell Biol* 1965; **25:** 407-408.

9. Reynolds ES. The use of lead citrate at high pH as an electron-opaque stain in electron microscopy. *J Cell Biol* 1963; **17:** 208-212.

10. Bissonette GB, Martins GJ, Franz TM, Harper ES, Schoenbaum G, Powell EM. Double dissociation of the effects of medial and orbital prefrontal cortical lesions on attentional and affective shifts in mice. *J Neurosci* 2008; **28**(44)**:** 11124-11130.

11. Hiramoto T, Kang G, Suzuki G, Satoh Y, Kucherlapati R, Watanabe Y *et al.* Tbx1: identification of a 22q11.2 gene as a risk factor for autism spectrum disorder in a mouse model. *Hum Mol Genet* 2011; **20**(24)**:** 4775-4785.

12. Ma Y, Hof PR, Grant SC, Blackband SJ, Bennett R, Slatest L *et al.* A three-dimensional digital atlas database of the adult C57BL/6J mouse brain by magnetic resonance microscopy. *Neuroscience* 2005; **135**(4)**:** 1203-1215.

**Figure S1.** High-resolution *ex vivo* fractional anisotropy (FA) image (150-μm isotropic voxel). The minimum deformation template was constructed from all b0 and scalar images (N = 17) and nonlinearly registered to the mouse atlas space ^12^. Each anatomical structure was used for statistical analyses.

**Figure S2.** (**a**) There were between-genotype differences in fractional anisotropy (FA) values in some regions (Genotype x Region, F(18, 270) = 2.795, p < 0.001). (**b)** Effect sizes of the FA differences between +/+ and +/- mice, as determined by Cohen’s d values. +/+, n = 10; +/-, n = 7.

**Figure S3. (a)**. Mann-Whitney U-tests revealed no between-genotype differences in axial diffusivity (AD) values in any of the regions examined (all p > 0.05). (**b)**. Effect sizes of the AD differences between +/+ and +/- mice, as determined by Cohen’s d values.

**Figure S4**. **(a)**. Mann-Whitney U-tests revealed no between-genotype differences in radial diffusivity (RD) values in any of the regions examined (all p > 0.05). (**b)**. Effect sizes of the RD differences between +/+ and +/- mice, as determined by Cohen’s d values.

**Figure S5.** **(a)**. Mann-Whitney U-tests revealed no between-genotype differences in mean diffusivity (MD) values in any of the regions examined (all p > 0.05). **(b)**. Effect sizes of the MD differences between +/+ and +/- mice, as determined by Cohen’s d values.

**Figure S6.** Representative images of the anterior (Bregma –0.82 to –1.33mm) and posterior (Bregma –1.34 to –1.94mm) regions of the fimbria and isthmus of the corpus callosum (CC). This anterior-posterior division was based on areas exhibiting significant reductions in fractional anisotropy (FA) signals without necessarily following the anatomical definition of the antero-posterior division. Gold staining intensity was measured in the demarcated areas. Sections of +/+ and +/- mice were mounted on the top and bottom rows, respectively, of each slide and stained in the Gold solution at the same time; some pairs were mounted in the opposite order.

**Figure S7.** Black-Gold II staining of myelin in the anterior fimbria (**a**), posterior fimbria (**b**), anterior isthmus of the corpus callosum (**c**), and posterior isthmus of the corpus callosum (**d**) (see **Materials and Methods**, **Black-Gold II staining**; **Supplemental Fig. S6**). Representative images of gold-stained myelin (left panels) and staining intensities of each pair of +/+ and +/- mice (right panels) are shown. As the assumption of normality was violated, data of the fimbria were analyzed by Wilcoxon signed rank tests. Neither the assumptions of normality nor homogeneity of variance was violated for data of corpus callosum, paired t-tests were used. +/- mice exhibited significantly decreased levels of gold staining in the anterior fimbria (*, p = 0.018), compared to +/+ mice. +/+ and +/- mice did not differ in the posterior fimbria (not significant (ns), p = 0.123), anterior corpus callosum (p = 0.310 or posterior corpus callosum (ns, p = 0.225). As these 4 tests were planned comparisons, no correction for multiple tests was applied. The staining intensity values of up to 8 anterior fimbriae and 10 posterior fimbriae from both hemispheres were used to compute the average of each mouse; those of up to 4 midline corpra callosa were used to compute the average of each mouse. Those paired cases were derived from 7 +/+ and 7 +/- mice for the anterior fimbria; 8 +/+ and 8 +/- mice for the posterior fimbria; 7 +/+ mice and 7 +/- mice for the anterior corpus callosum; 5 +/+ and 5 +/- mice for the posterior corpus callosum. Scale bar = 200 μm.

F**igure S8.** Average (± standard error of the mean [SEM]) myelin thickness at each 100-nm axon diameter unit. **a)** An overall generalized linear mixed model (GLMM) analysis shows a significant interaction between the genotype and axon diameter interval in the 200 -1600 nm range where myelinated axons were detected in the fimbria of both +/+ and +/- mice (F (14,454) = 2.280, p = 0.005). Applying an exploratory GLMM to the data of 100 nm axon diameter units from 200 - 600 nm and 700 - 1600nm ranges which showed clearly different patterns, we demonstrate that the significant interaction is due to thicker myelin in axons diameters from 700 -1600 nm in the +/- mice than in the +/+ mice (genotype, F (1,210) = 27.649, p < 0.001; interaction, F (9, 210) = 0.901, p = 0.526). No difference is detected in the 200 - 600 nm range (genotype, F (1,244) = 0.473, p = 0.492; interaction, F (4,244) = 0.528, p = 0.715). **(b)** An overall GLMM analysis shows a significant interaction between the genotype and axon diameter interval (F (17,421) = 2.918, p < 0.001) in the axon diameter range of 200-1900 nm where myelinated axons were detected in the corpus callosum of both the +/+ and +/- mice. Exploratory GLMM’s of the data of 100 nm axon diameter units from 200 - 900 nm and 1000 - 1300 nm ranges demonstrate that the significant interaction was due to thinner myelin in axons of diameter 1000 -1300 nm in the +/- mice than in the +/+ mice (genotype, F (1,55) = 6.792, p = 0.012; genotype x interval, F (3,55) = 0.632, p = 0.598). No difference was detected in the 200 - 900 nm range (genotype, F(1,351) = 0.126, p = 0.723; interaction, F (7,351) = 0.821, p = 0.570). Between 1400 and 1900 nm there were only one case per a 100 nm unit (i.e., no variance), and thus, no GLMM was applied to that range.

**Figure S9.** The proportion of myelinated axons at each 100 nm axon diameter unit. **a**) The relative proportion of myelinated axons in the fimbria with diameters > 500 nm - < 1,200 nm was higher in the +/- mice than in the +/+ mice, while that of axons with diameters > 1,200 nm was lower. No axons with diameters equal to or larger than 1,700 nm were observed in the fimbria (**Table S1a**, Fimbria). **(b)** In the corpus callosum, the relative proportion of myelinated axons with diameters > 400 nm - < 800 nm was higher in the +/- mice than in the +/+ mice, while that of axons with diameters equal to or larger than > 800 nm was lower (see also **Table S1b**, Corpus callosum). Values are expressed as percentages [(# of axons in each unit / # of all axons in region) x 100].

**Figure S10.** Olfactory responses to non-social and social odorants. The mean (± standard error of the mean [SEM]) sniffing time (s) at an Eppendorf tube containing each odorant. The assumptions of normality and homogeneity of variance were violated, as assessed using Shapiro–Wilk tests and Levene’s tests, respectively. Non-parametric Mann–Whitney U-tests, adjusted by Benjamini–Hochberg’s correction, revealed no differences between +/+ and +/- mice for any odorant in any session. W, water; A, almond odor; B, banana odor; NL, urine of non-littermate C57BL/6J male mouse; +/-, urine of a non-littermate +/- female mouse; rm, urine of the dam of tested mice, am, urine of a non-dam mother; v; urine of a virgin female C57BL/6 mouse. +/+, N = 14 and +/-, N = 14 for water; almond and banana, NL1, NL2, and HT. +/+, N = 26, +/-, N = 24 for rm and am, +/+, N = 9, +/-, N = 11 for v.

**Table S1**. Number and percentage of axons in each 100nm axon diameter range (nm)

1. **Fimbria**

| Genotype | | Axon diameter (nm) | | | | | | | | | | | | | | | | Total (n) |
| --- | --- | --- | --- | --- | --- | --- | --- | --- | --- | --- | --- | --- | --- | --- | --- | --- | --- | --- |
|  |  | <300 | 300 | 400 | 500 | 600 | 700 | 800 | 900 | 1000 | 1100 | 1200 | 1300 | 1400 | 1500 | 1600 | ≥1700 |  |
| +/+ | n | 7 | 24 | 47 | 31 | 29 | 37 | 26 | 14 | 15 | 10 | 10 | 12 | 8 | 6 | 8 | 16 | 300 |
|  | % | 2.3 | 8.0 | 15.7 | 10.3 | 9.7 | 12.3 | 8.7 | 4.7 | 5.0 | 3.3 | 3.3 | 4.0 | 2.7 | 2.0 | 2.7 | 5.3 |  |
| +/- | n | 7 | 20 | 30 | 31 | 28 | 17 | 16 | 18 | 11 | 7 | 4 | 5 | 2 | 2 | 2 | 0 | 200 |
|  | % | 3.5 | 10 | 15 | 15.5 | 14 | 8.5 | 8 | 9 | 5.5 | 3.5 | 2 | 2.5 | 1 | 1 | 1 | 0 |  |

1. **Corpus Callosum**

| Genotype | |  | | Axon diameter (nm) | | | | | | | | | | | | | | | | | | Total (n) |
| --- | --- | --- | --- | --- | --- | --- | --- | --- | --- | --- | --- | --- | --- | --- | --- | --- | --- | --- | --- | --- | --- | --- |
|  |  | <300 | 300 | | 400 | 500 | 600 | 700 | 800 | 900 | 1000 | 1100 | 1200 | 1300 | 1400 | 1500 | 1600 | 1700 | 1800 | 1900 | ≥2000 |  |
| +/+ | n | 6 | 31 | | 35 | 37 | 29 | 26 | 25 | 10 | 10 | 12 | 10 | 10 | 5 | 4 | 1 | 3 | 1 | 2 | 3 | 260 |
|  | % | 2.3 | 11.9 | | 13.5 | 14.2 | 11.2 | 10.0 | 9.6 | 3.8 | 3.8 | 4.6 | 3.8 | 3.8 | 1.9 | 1.5 | 0.4 | 1.2 | 0.4 | 0.8 | 1.2 |  |
| +/- | n | 2 | 21 | | 32 | 41 | 26 | 28 | 11 | 7 | 2 | 9 | 9 | 1 | 1 | 5 | 2 | 1 | 1 | 1 | 0 | 200 |
|  | % | 1.0 | 10.5 | | 16 | 20.5 | 13 | 14 | 5.5 | 3.5 | 1 | 4.5 | 4.5 | 0.5 | 0.5 | 2.5 | 1 | 0.5 | 0.5 | 0.5 | 0 |  |

The relative numbers of myelinated axons differed between the +/+ and +/- mice in the fimbriae (**a,** χ^2^(15, N=500) = 27.314, p=0.026), but not in the corpus callosum (**b,**  χ^2^(18, N=460) = 23.673, p=0.166). Each column is a 100 nm unit of axon thickness (e.g., 300; >300 to <400) except for the first (<300nm) and last (a. >1700 columns; b, >2000). This overall difference in the fimbria was primarily due to the apparent absence of large myelinated axons (>1,700 nm) in the +/- mice (**Figure S9a**). Analyzing the >1700 nm unit poses a technical problem in running an exploratory χ^2^ test due to a zero value in +/- mice. We, therefore, analyzed the relative numbers of axons by combining the >1600 – < 1700 nm units and > 1700 nm units. The +/- mice had significantly fewer large myelinated axons in this range than the +/+ mice (χ^2^(1, N=26) = 11.308, p = 0.001). Fimbria, +/+, N = 300, +/-, N = 200; Corpus callosum, +/+, N = 260, +/-, N = 200.

|  |  |  |
| --- | --- | --- |
|  | **Table S2. Intensity calculations** |  |
| **Step** | **Value** | **Formula** |
| 1 | Brightness of target | tB/A |
| 2 | Brightness of negative control | ncB/A |
| 3 | Ratio of each negative control | (max ncB/A)/(ncB/A)=R |
| 4 | Adjusted brightness | tN/A^adj^=(tB/A)*R |
| 5 | Conversion to staining intensity | 1/(tB/A^adj^) |

B, Brightness of the target (t) region; A, Area of the target region; tB/A, brightness

per target area; ncB/A, Brightness per area unit of the cortex as a negative control (nc)

of non-specific staining; R, the corrective ratio for adjustment based on the intensity of

non-specific staining. The brightest cortical section (max ncB/A) was considered to be devoid of non-specific staining. Cortical areas exhibiting less brightness (<max ncB/A) were considered to exhibit a degree of non-specific staining, resulting in R values larger than 1.0. If tB/A is multiplied by R, the adjusted brightness value ((tB/A)*R ) is corrected proportional to the degree of non-specific staining. As this value is negatively proportional to the degree of gold staining, we used its inverse value to represent the degree of gold staining. Finally, the 5^th^ value was multiplied by 10^3^ for fimbria and 10^2^ for corpus callosum, so that both sets of values had at least one natural number before the decimal point. Comparison was made between +/+ and +/- within each brain region.

| \| **Table S3. Assay ID numbers** \| \| \| --- \| --- \| \| **Gene Symbol** \| **Assay ID** \| \| *Tbx1 exon 2-3* \| Mm01342798_m1 \| \| *Ng2 (Cspg4)* \| Mm00507257_m1 \| \| *Pdgfr2* \| Mm00440701_m1 \| \| *MBP* \| Mm01266402_m1 \| \| *MOG* \| Mm01279062_m1 \| \| *Cyc1* \| Mm00470540_m1 \| \|  \|  \| |  |
| --- | --- | --- | --- | --- | --- | --- | --- | --- | --- | --- | --- | --- | --- | --- | --- | --- | --- | --- | --- |

**Table S4. Dimensions and exemplar combinations used for attentional set shifting**

| Task | Dimension | | Exemplar combinations | |
| --- | --- | --- | --- | --- |
|  | Relevant | Irrelevant | Correct | Incorrect |
| SD  CD    IDS I    IDS II    IDS III    IDS IV    IDS IV rev  EDS | Odor (O) Medium (M)  Odor    Odor  Odor    Odor    Odor  Odor  Medium | ---------    Medium  Medium  Medium  Medium  Medium  Medium  Odor | O1&M1  O1&M1  O1&M2  O3&M3  O3&M4  O5&M5  O5&M6  O7&M7  O7&M8  O9&M9  O9&M10  O10&M9  O10&M10  M11&O11  M11&O12 | O2&M2  O2&M2  O2&M1  O4&M4  O4&M3  O6&M6  O6&M5  O8&M8  O8&M7  O10&M10  O10&M9  O9&M10  O9&M9  M12&O12  M12&O11 |

See **Table S5** for each exemplar. SD, simple discrimination; CD, compound discrimination;

IDS, intra-dimensional shift; rev, reversal; EDS, extradimensional shift.

**Table S5: Odorants and media used as exemplars of attentional set shifting**

| **Pair** | **Test** | **Exemplar** | **Odor** | **Medium** |
| --- | --- | --- | --- | --- |
| 1 | SD, CD | 1 | Sage（O1） | Alpha drip（M1） |
|  |  | 2 | Cinnamon （O2） | Paper chip（M2） |
| 2 | IDS I | 3 | Coriander （O3） | Carefresh Natural（M3） |
|  |  | 4 | Onion （O4） | Kaykob bedding（M4） |
| 3 | IDS II | 5 | Garlic （O5） | Eco bedding（M5） |
|  |  | 6 | Paprika （O6） | Sphang moss（M6） |
| 4 | IDS III | 7 | Rosemary （O7） | Aspen Bedding（M7） |
|  |  | 8 | Cloves （O8） | Aquarium Gravel（M8） |
| 5 | IDS IV, IDSIV rev | 9 | Thyme （O9） | Rapti bark（M9） |
|  |  | 10 | Black Pepper（O10） | Shredded paper（M10） |
| 6 | EDS | 11 | Cumin （O11） | ExquisiCat（M11） |
|  |  | 12 | Cardamom（O12） | Carefresh Ultra（M12） |

SD, simple discrimination; CD, compound discrimination; IDS, intra-dimensional shift;

rev, reversal; EDS, extradimensional shift.
