# Supplementary figures and images for: Tbx1, a gene encoded in 22q11.2 copy number variant, is a link between alterations in fimbria myelination and cognitive speed in mice
Source: Mol Psychiatry. 2021 Nov 5;27(2):929–38. doi: 10.1038/s41380-021-01318-4 (PMC9054676; doi:10.1038/s41380-021-01318-4)

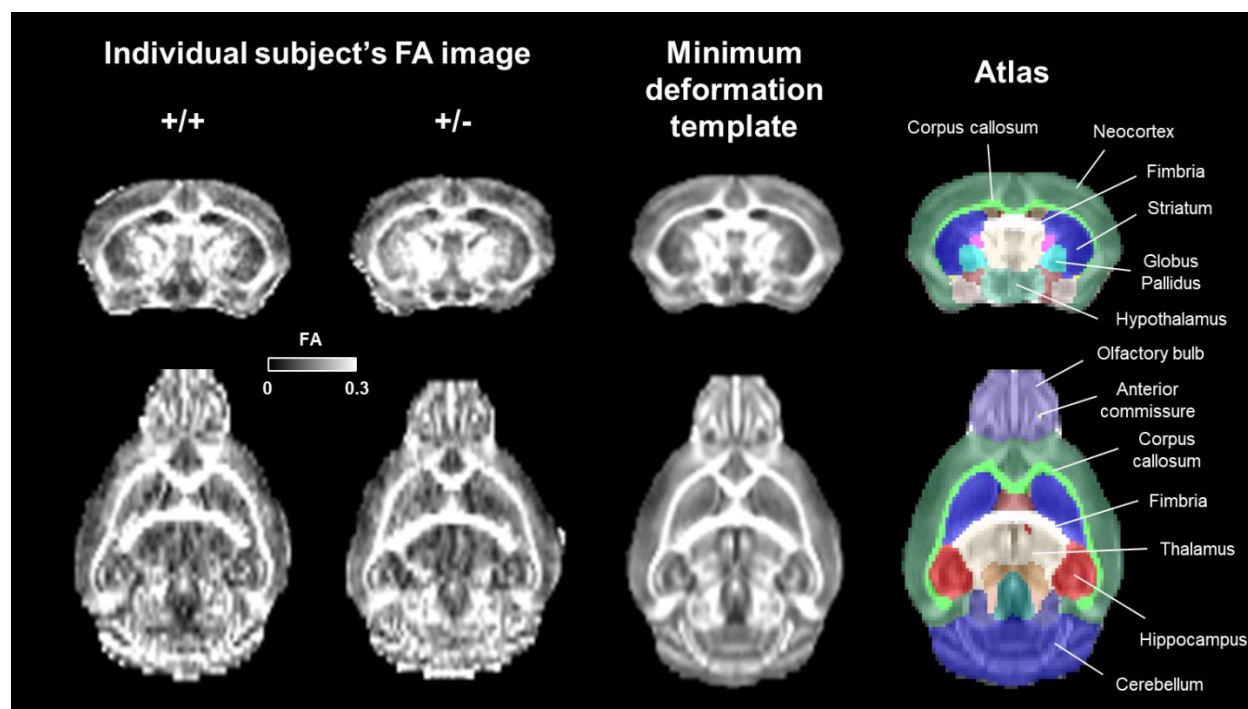

Figure S1.

Supplement: Supplementary file 2 — Supplementary Figure 1 [file 41380_2021_1318_MOESM2_ESM.pdf]

**a**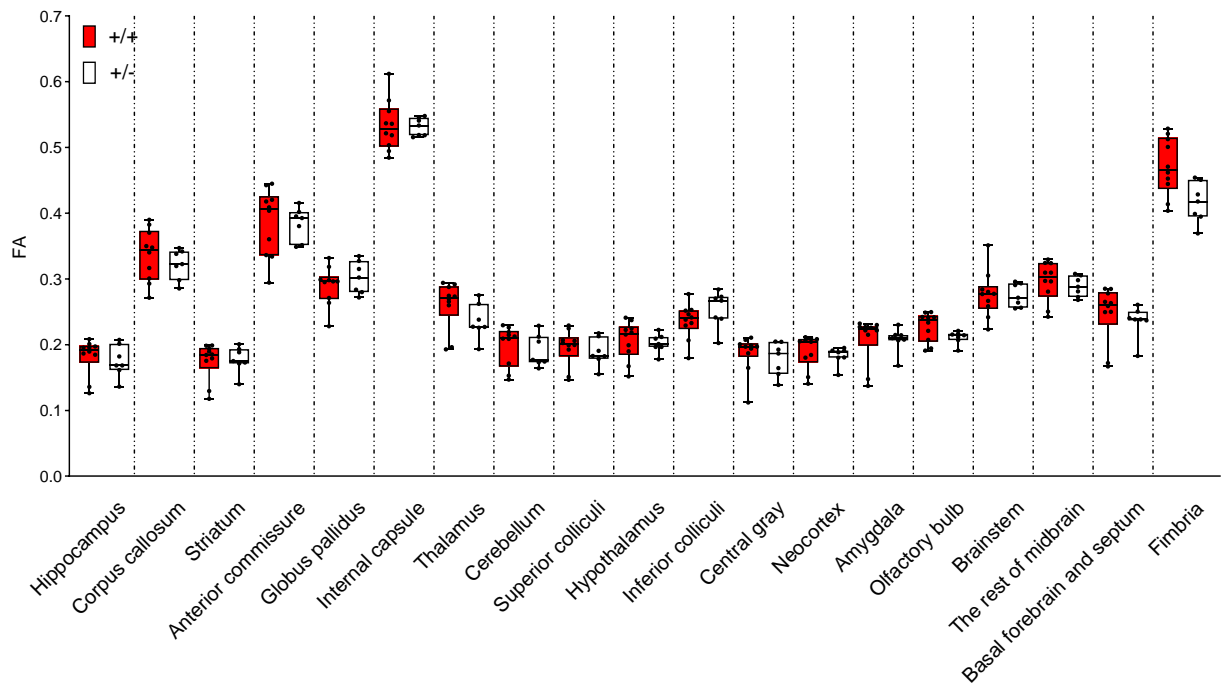**b**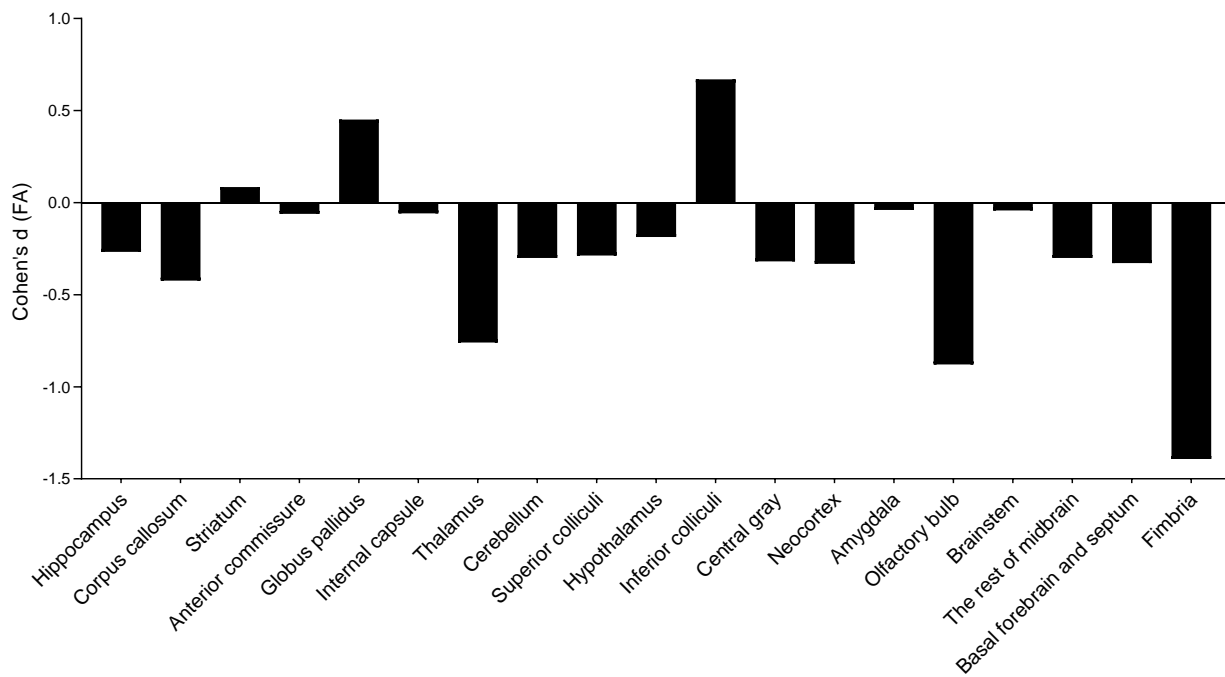**Figure S2.**

Supplement: Supplementary file 3 — Supplementary Figure 2 [file 41380_2021_1318_MOESM3_ESM.pdf]

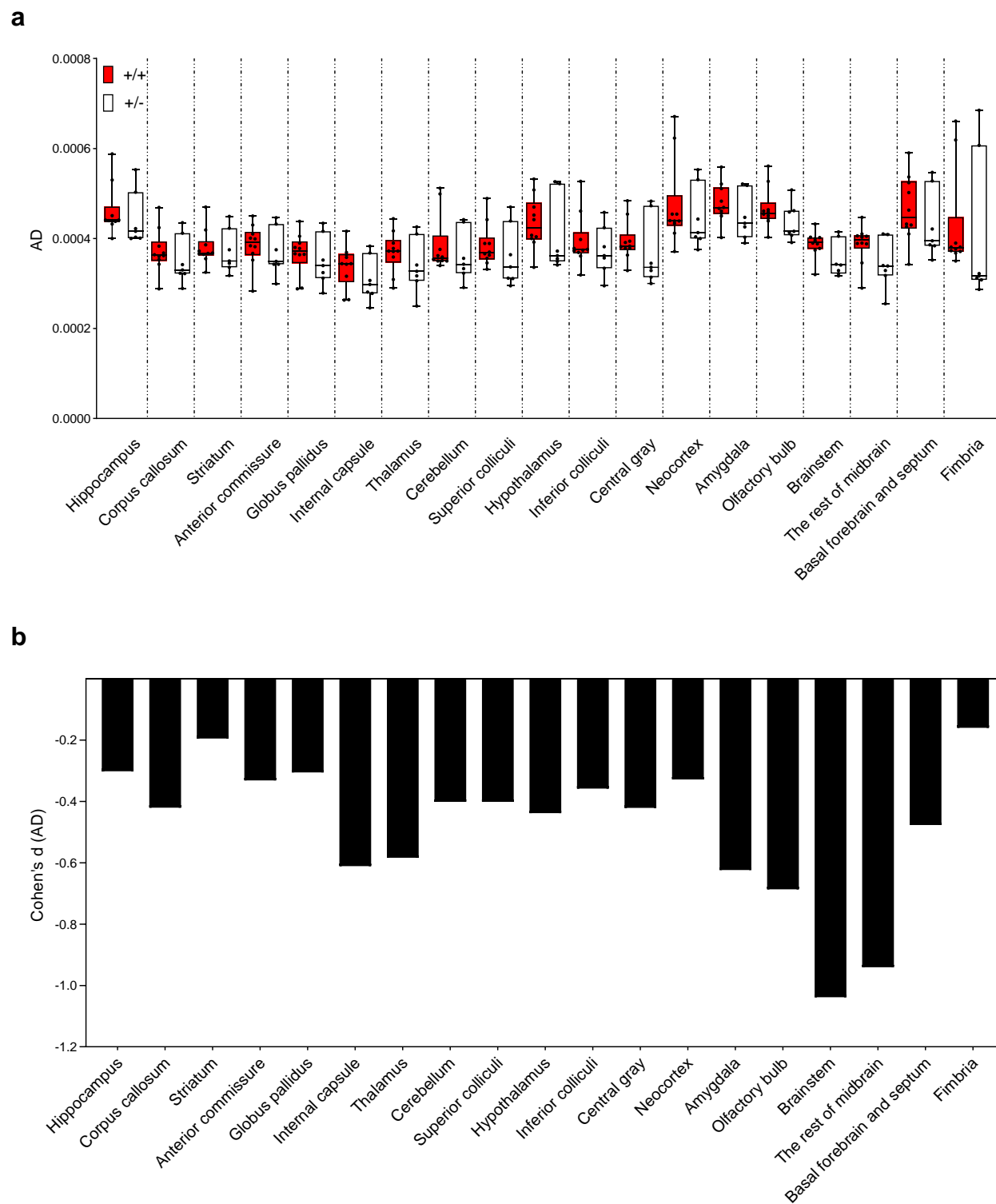

**Figure S3.**

Supplement: Supplementary file 4 — Supplementary Figure 3 [file 41380_2021_1318_MOESM4_ESM.pdf]

**a**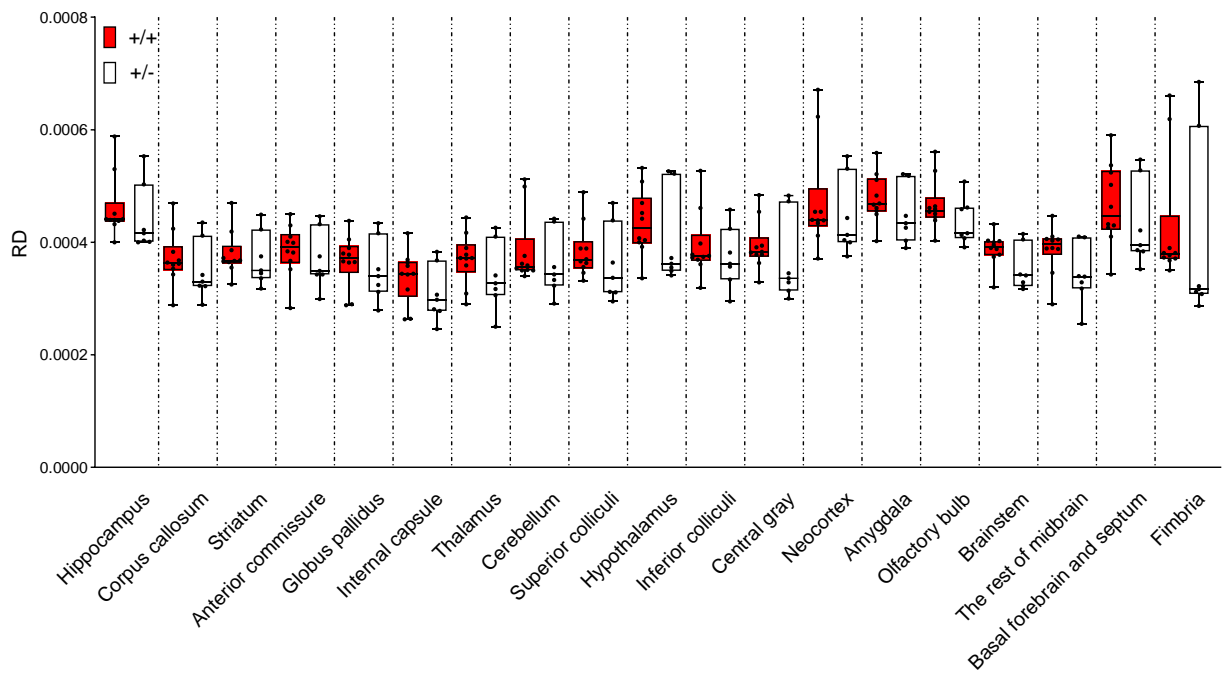**b**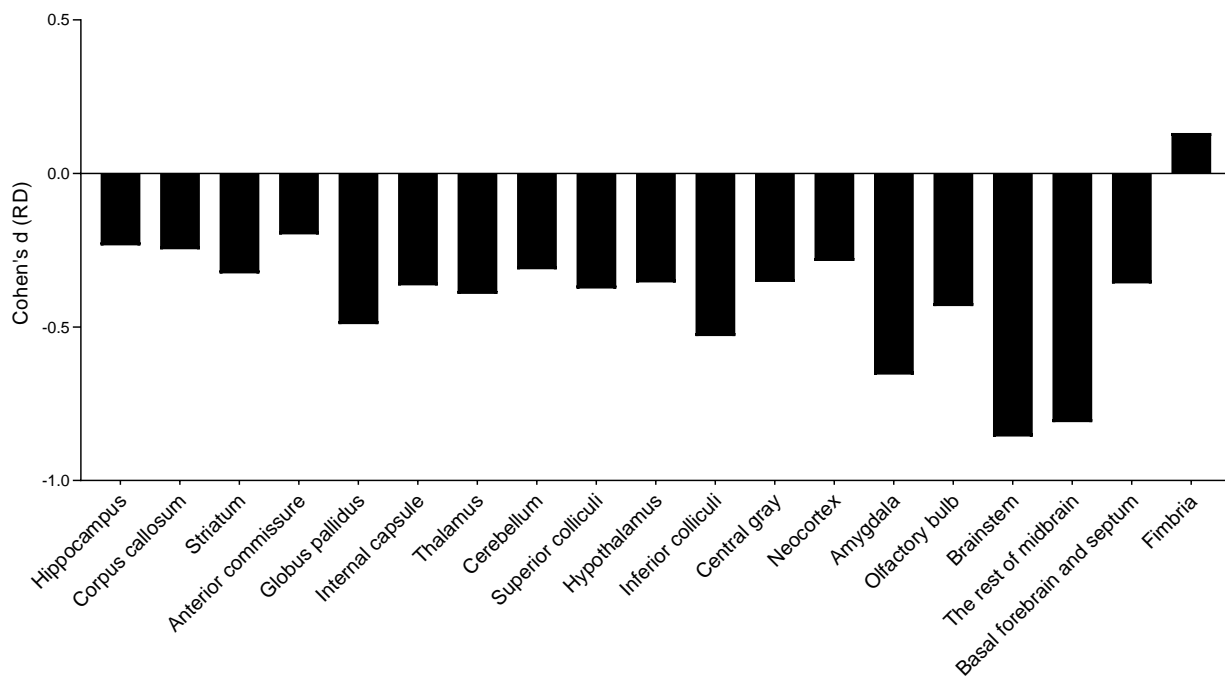**Figure S4.**

Supplement: Supplementary file 5 — Supplementary Figure 4 [file 41380_2021_1318_MOESM5_ESM.pdf]

**a**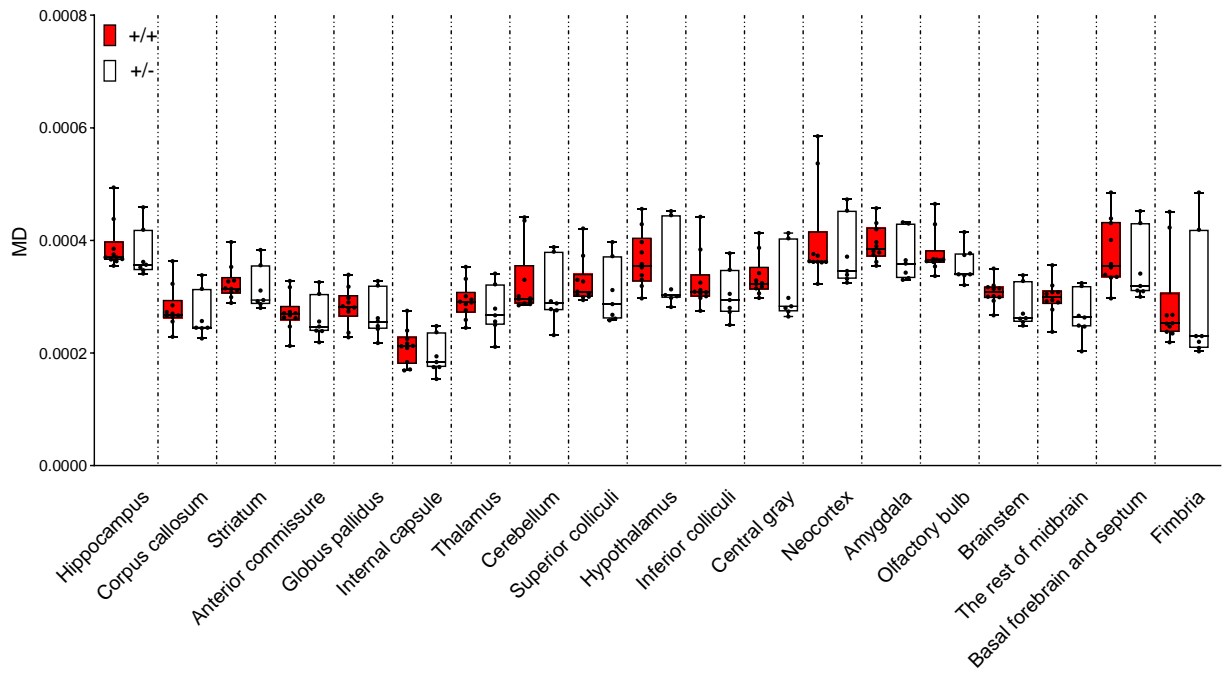**b**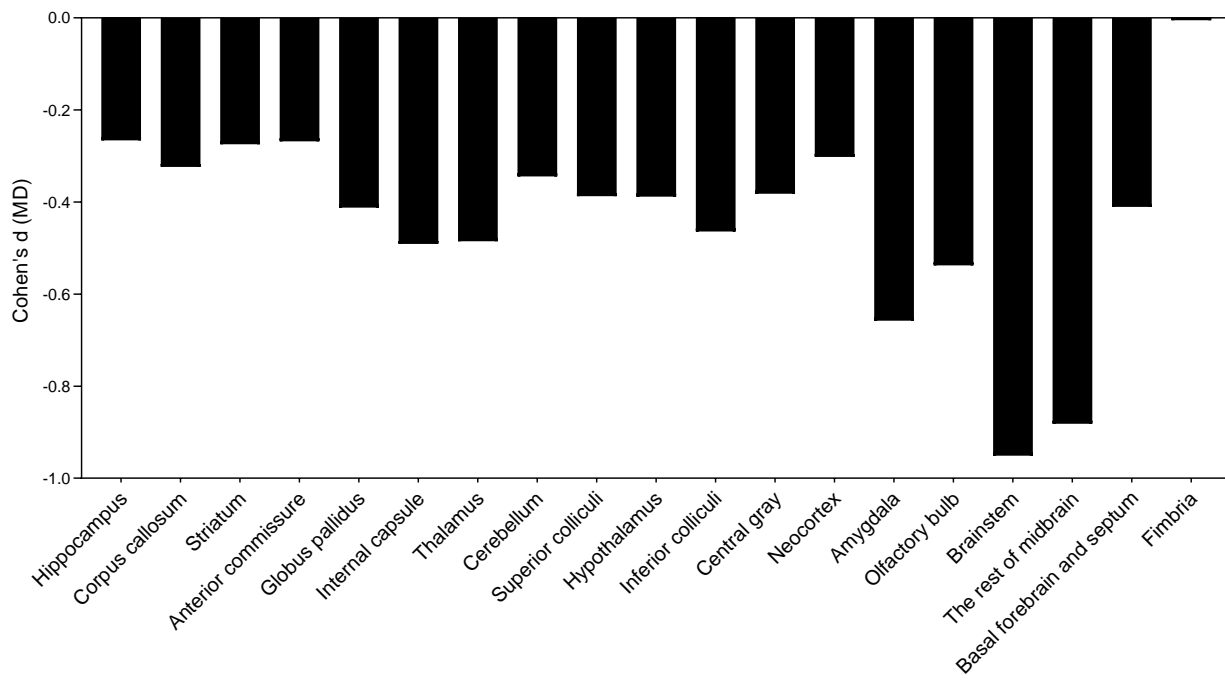**Figure S5**

Supplement: Supplementary file 6 — Supplementary Figure 5 [file 41380_2021_1318_MOESM6_ESM.pdf]

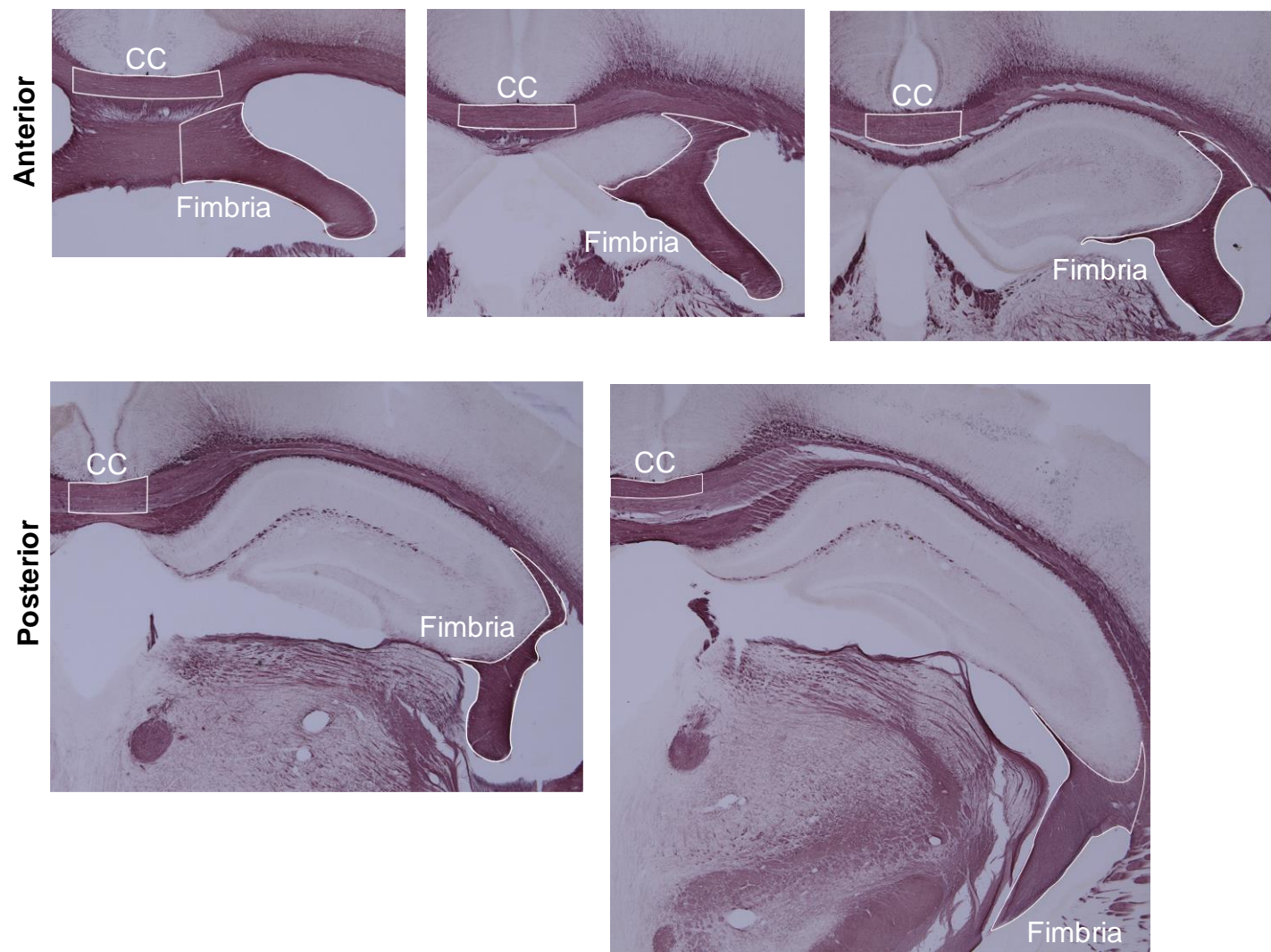

**Figure S6.**

Supplement: Supplementary file 7 — Supplementary Figure 6 [file 41380_2021_1318_MOESM7_ESM.pdf]

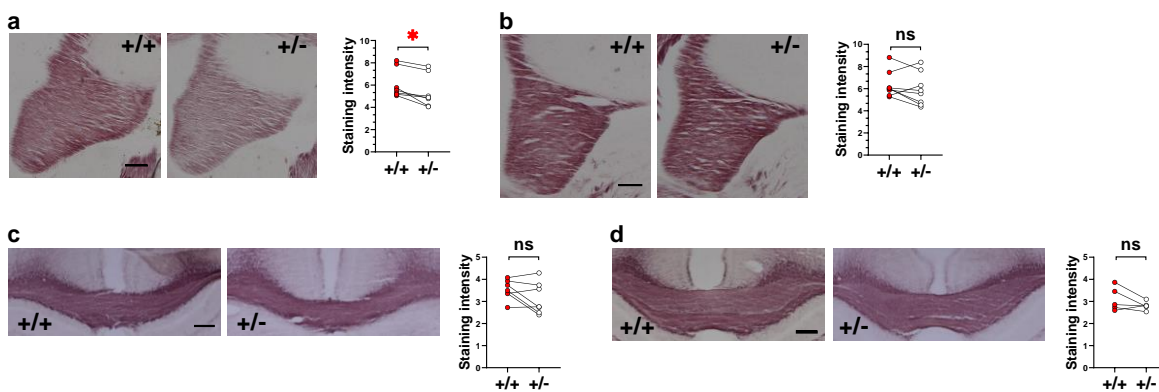

**Figure S7.**

Supplement: Supplementary file 8 — Supplementary Figure 7 [file 41380_2021_1318_MOESM8_ESM.pdf]

a

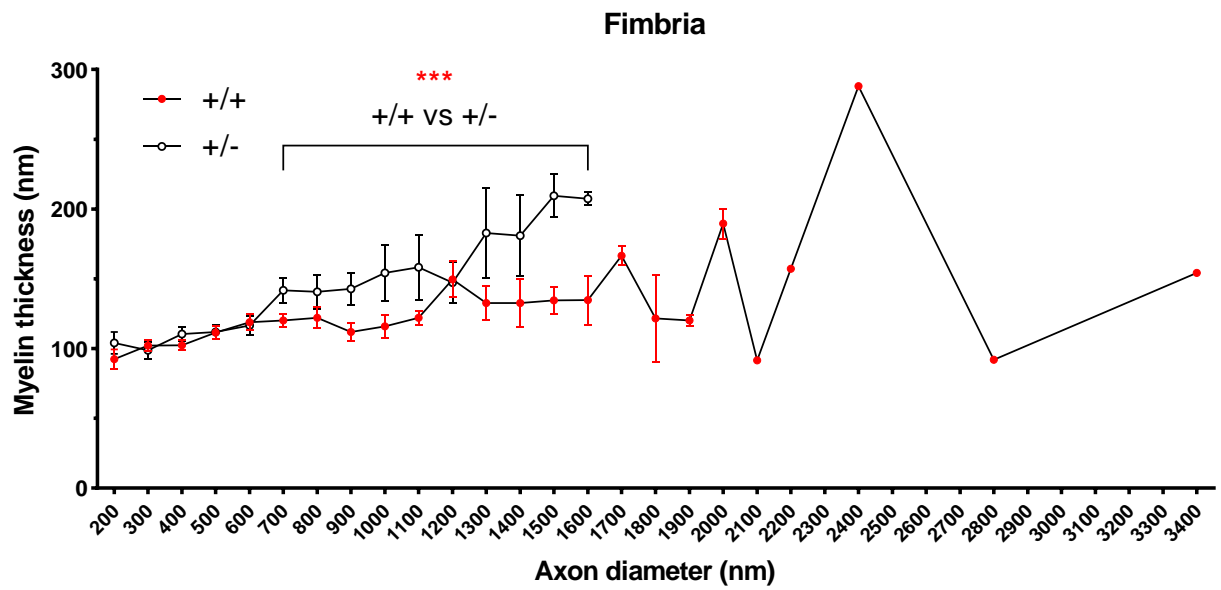

b

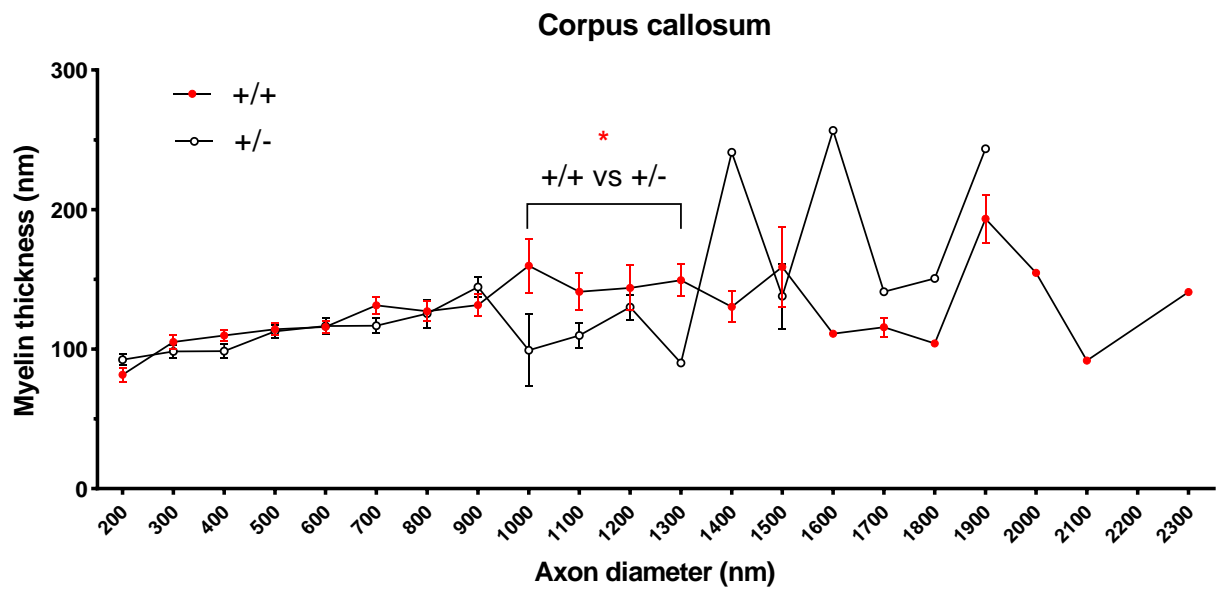

Figure S8.

Supplement: Supplementary file 9 — Supplementary Figure 8 [file 41380_2021_1318_MOESM9_ESM.pdf]

**a**

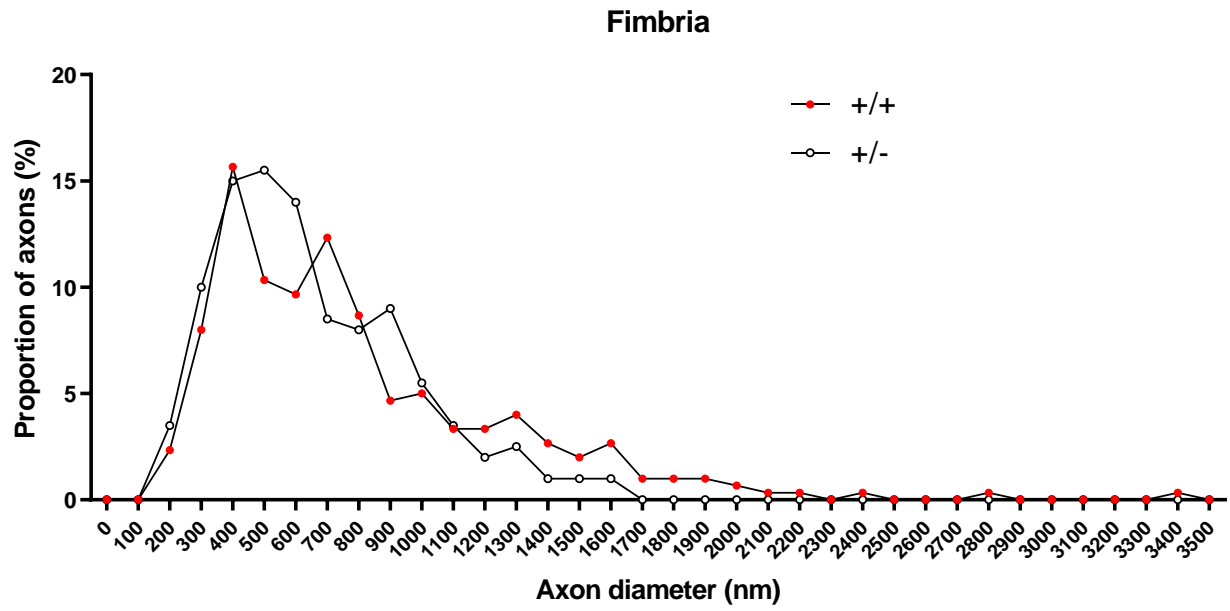

**b**

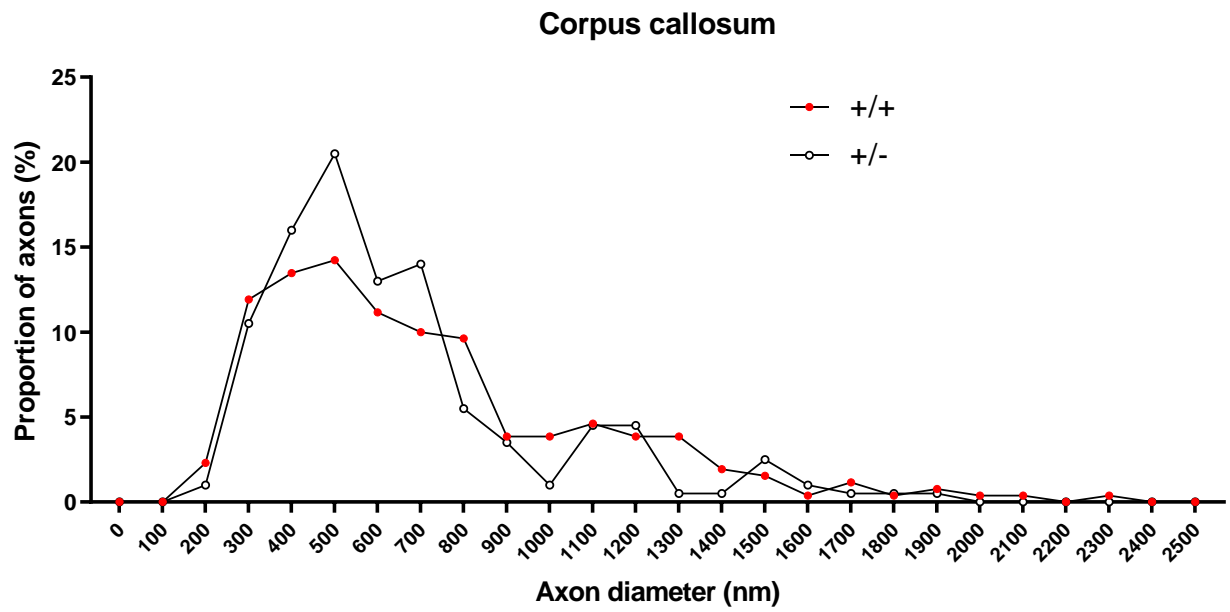

**Figure S9 .**

Supplement: Supplementary file 10 — Supplementary Figure 9 [file 41380_2021_1318_MOESM10_ESM.pdf]

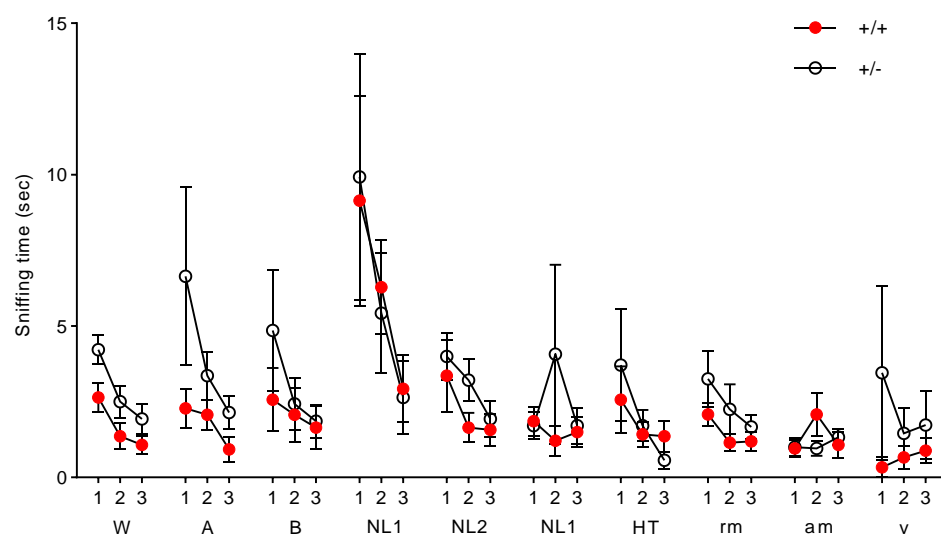

**Figure S10.**

Supplement: Supplementary file 11 — Supplementary Figure 10 [file 41380_2021_1318_MOESM11_ESM.pdf]
